# Higher isokinetic quadriceps peak force is associated with a patient‐acceptable symptom‐state 1 and 3 years after ACL reconstruction

**DOI:** 10.1002/ksa.12541

**Published:** 2024-11-20

**Authors:** Farshad Ashnai, Roland Thomeé, Eric Hamrin Senorski, Susanne Beischer

**Affiliations:** ^1^ Department of Orthopaedics, The Institute of Clinical Sciences at Sahlgrenska Academy University of Gothenburg Gothenburg Sweden; ^2^ Sportrehab Sports Medicine Clinic Gothenburg Sweden; ^3^ Department of Health and Rehabilitation, Unit of Physiotherapy, The Institute of Neuroscience and Physiology, Sahlgrenska Academy University of Gothenburg Gothenburg Sweden; ^4^ Department of Orthopaedics Sahlgrenska University Hospital Mölndal Sweden

**Keywords:** orthopaedics, physical therapy, reference values, rehabilitation, sports medicine

## Abstract

**Purpose:**

The main purpose was to determine cut‐off values for absolute (Q_Nm/kg_) and relative (Q_LSI_) isokinetic knee extensor (KE) strength for achieving a patient‐acceptable symptom state (PASS) in the Knee injury and Osteoarthritis Outcome Score (KOOS) subscales and for different age groups to determine the association between Q_Nm/kg_ and Q_LSI_ and PASS, at 1 and 3 years after an anterior cruciate ligament reconstruction (ACLR).

**Methods:**

PASS was defined as reaching cut‐off values for all KOOS subscales. Data from follow‐ups were extracted from a rehabilitation registry. Male and female patients were divided into two age groups based on their age at primary ACLR: 16–24 years and 25–65 years. Odds Ratios between the Q_Nm/kg_ and Q_LSI_ cut‐off values and achieving PASS were calculated. Receiver Operating Characteristic curves were constructed to determine the individual predictive capacity for achieving PASS of Q_Nm/kg_ and of Q_LSI_ using the area under the curve (AUC).

**Results:**

Results from 755 and 145 patients (females = 51% and 52%; preinjury Tegner Activity level ≥6 = 82% and 74%) were used in the 1‐ and 3‐year follow‐up analyses. Reaching the cut‐off values for the Q_Nm/kg_, ranging between ≥2.1 and ≥2.7, entailed between 2.09 and 5.12 times the odds of achieving PASS, across all groups at the 1‐year follow‐up. At the 3‐year follow‐up, the cut‐off values of ≥3.4 and ≥2.6Q_Nm/kg_ were associated with patients achieving PASS with acceptable accuracy (AUC = 0.700–0.780) in 16–41 year‐old males and females.

**Conclusion:**

At 1 year after ACLR, patients of both sexes and age groups reaching cut‐off values for absolute KE strength had two to five times the odds, that were clinically relevant, to achieve PASS. Acceptable discriminative capacity was found for the absolute KE strength among male and female patients 16–24 years old, at 3 years after ACLR.

**Level of Evidence:**

Level III.

**Abbreviations**
ACL: anterior cruciate ligament
ACLR: anterior cruciate ligament reconstruction
ADL: activities of daily living
AUC: area under the curve
KE: knee extensor
KOOS: Knee injury and Osteoarthritis Outcome Score
LSI: limb symmetry index
Nm: Newton metre
OR: odds ratio
PASS: patient‐acceptable symptom state
PROM: patient related outcome measure
Q_LSI_
: (peak torque of the ACL‐reconstructed limb/peak torque of the healthy limb) * 100
Q_Nm/kg_
: peak torque of the ACL‐reconstructed limb/body weight
QoL: quality of life

## INTRODUCTION

An anterior cruciate ligament (ACL) injury is a severe knee injury that results in a long period of rehabilitation and a reduction in physical activity [1]. One consequence of the injury is strength deficits in the knee extensors (KE) [16], due partly to pain and partly to unloading of the injured limb [7]. Deficits of between 5% and 27% in KE strength have been reported at 1 year after ACL reconstruction (ACLR) [16], which might be problematic, as patients with lower KE strength symmetry report poorer knee‐related function at 7, 12 and 24 months after ACLR [5, 11, 22]. Furthermore, more symmetrical KE strength has been reported to reduce the risk of a subsequent traumatic knee injury [8] and the development of symptomatic, as well as radiographic knee osteoarthritis [15]. Therefore, restoring patients' KE strength is an important rehabilitation goal for all patients and all physical therapists, to lessen the long‐term consequences after ACL injury.

The Knee injury and Osteoarthritis Outcome Score (KOOS) [21] is widely used to evaluate patients' perceived pain, symptoms, activities of daily living (ADL), function in sports and recreation (sports) and knee‐related quality of life (QoL) after ACLR [24]. The patient‐acceptable symptom state (PASS) of the KOOS [14] provides an average value at which patients consider themselves well (answering yes on a single question, described in the Methods), and the PASS is regarded as an important complementary tool in helping clinicians interpret the results from the KOOS [28].

The results of tests of muscle strength are commonly reported as the Limb Symmetry Index (LSI), and it is calculated as the results for the injured limb divided by the result for the noninjured limb and expressed as a percentage. However, absolute isometric KE strength in Newton metres (Nm) divided by body weight (Q_Nm/kg_) has been reported to be a stronger predictor of high self‐reported knee function compared with the LSI (Q_LSI_) 3 years after ACLR [18]. The corresponding cut‐off values for isokinetic KE strength at 1 and 3 years after ACLR have not been described in the literature. Cut‐off values for isokinetic KE strength may be valuable for patients and physical therapists to aim for in rehabilitation following ACLR to facilitate the achievement of acceptable knee function.

The purpose of this study was to determine cut‐off values for the Q_Nm/kg_ and Q_LSI_ for achieving a PASS through predefined cut‐off values in the KOOS subscales (henceforth referred to as PASS) and to determine, for different age groups, the association between the Q_Nm/kg_ and Q_LSI_ and PASS at 1 and 3 years after ACLR, respectively.

A further purpose was to present reference values, for different age groups, of the peak isokinetic KE strength and LSI during the first 5 years (in this study at 10 weeks, 4, 8, 12 and 18 months and 2, 3 and 5 years) following ACLR.

We hypothesized that both the Q_Nm/kg_ and Q_LSI_ would be associated with the achievement of PASS for the KOOS subscales. In addition, we hypothesized that the Q_Nm/kg_ would have a better capacity to discriminate between patients who achieved and did not achieve PASS for the KOOS subscales compared with the Q_LSI_, 1 and 3 years postoperatively.

## METHODS

To improve transparency, this cross‐sectional diagnostic study was reported using the Standards for Reporting Diagnostic Accuracy statement as a checklist [3].

This study was based on data extracted from Project ACL, an ongoing local rehabilitation registry established in the western part of Sweden in 2014, consisting of data from patients with an ACL injury. Participation in Project ACL is voluntary, and all the data are collected after informed consent. Ethical approval was obtained from the Regional Ethical Review Board in Sweden (registration numbers: 265‐13, T023‐17) and the Swedish Ethical Review Authority (registration number: 2020‐02501). In Project ACL, patients are regularly evaluated with five tests of physical performance and a series of patient‐reported outcome measures (PROM) preoperatively, at 10 weeks, 4, 8, 12 and 18 months and 2, 3 and 5 years after injury and/or ACLR and every 5th year thereafter. Patients are invited to participate in the registry at any time point following an ACL injury. For example, patients may be evaluated at 12 months after undergoing ACLR without having been evaluated within the registry before. In this study, the results of a test of maximum KE strength and the KOOS were used for analysis. In addition, data regarding patients' sex, age at primary ACLR, height, weight, body mass index, physical activity [24, 25] and graft harvesting site were extracted from evaluations performed at 10 weeks, 4, 8, 12 and 18 months and 2, 3 and 5 years after ACLR between 5 December 2015 and 7 November 2021.

### Patients

Patients aged 16–65 years at primary ACLR, who had responded to the KOOS and had performed tests of KE strength at the respective follow‐up at 10 weeks to 5 years after ACLR were included. Data from patients who sustained a new ACL injury in the same or contralateral limb within 5 years after the primary ACL injury were included if data were available from follow‐ups before the second ACL injury. Furthermore, patients with other injuries, in the same or the contralateral limb, and patients with symptoms such as pain and swelling superseding expected levels (i.e., reporting a swelling or pain >3/10 on a Numerical Rating Scale on the day of testing, or based on the judgement of the examiner), or with the presence of other nonknee‐related diseases (e.g., seasonal influenza), or medical status such as a pregnancy were excluded if this was thought to affect the result of KE strength testing or the rehabilitation.

### Knee extension strength

Concentric isokinetic KE strength was measured unilaterally, with the ACL‐injured limb tested first, followed by the contralateral limb. The tests were conducted by registered physical therapists educated in the test procedure, which has previously been described in detail [2]. In short, the test was performed with the patient seated in a stationary dynamometer, Biodex System 4 (Biodex Medical Systems, Inc.), with the range of motion set between 0^o^ and 90^o^ of knee flexion. The angular velocity was set at 90°/s and the peak torque was described in Nm. Patients performed a standardized warm‐up routine including 10 min of stationary cycling, 10 repetitions at ≈50% of one repetition maximum (RM), followed by 10 reps at ≈75% 1RM and one to two trial reps at ≈90% of 1RM before performing three maximum contractions, each separated by 40 s of rest. The best result for each limb was used for further analysis and was expressed in two ways:

‐ Absolute Muscle Strength (Q_Nm/kg_): peak torque of the ACL‐reconstructed limb/body weight
‐ Q_LSI_: (peak torque of the ACL‐reconstructed limb/peak torque of the healthy limb) * 100.

### Patient‐reported outcomes

#### KOOS

The KOOS is a 42‐item questionnaire with questions related to the knee, divided into five subscales: *Pain*, *Symptoms*, *ADL*, *Function in Sports and Recreation* (Sports), and *Knee‐related QoL*. The questions are answered on a 5‐level Likert scale, were 0 = no problems and 4 = extreme problems. The score from each subscale is calculated and likewise presented independently. The mean score for the questions in a particular subscale is divided by 4 and multiplied by 100. This number is then subtracted from 100 and represents the KOOS subscale estimate where 0 = extreme symptoms and 100 = no symptoms.

The Swedish version of the KOOS has been shown to be a reliable and valid PROM for assessing knee‐related symptoms, function and QoL in patients with ACL injury [4]. In Project ACL, the KOOS is completed at all follow‐ups as the strength tests are performed and, for this study, data were extracted from the evaluations performed at the 1‐year follow‐up to reflect the time point at which most patients have attempted to return to sport [12]. Furthermore, data were extracted from the evaluations performed at the 3‐year follow‐up to reflect a time point when patients can be expected to have terminated their standard rehabilitation period [12]. The KOOS has been reported to have acceptable test–retest reliability for patients with a knee injury (ICC = 0.85–0.93) [21].

The achievement of PASS was assessed by using the threshold values reported for patients with an ACL injury by Muller et al. [14] and defined in the current study as achieving the threshold values for each of the five subscales of the KOOS; Pain > 88.9, Symptoms > 57.1, ADL = 100, Sports > 75.0 and QoL > 62.5. Patients who reached the threshold values in fewer than five subscales were defined as not achieving the PASS. The PASS values have been reported to have moderate to high specificity (0.67–0.89) and sensitivity (0.70–0.87) [14]. The values have been based on the question ‘Taking into account all the activity you have during your daily life, your level of pain, and also your activity limitations and participation restrictions, do you consider the current state of your knee satisfactory?’ in a cohort of ACL‐reconstructed patients [14].

### Statistical analysis

Statistical analyses were conducted using the SAS statistical analysis system 169 (SAS/STAT Version 14.2; SAS Institute Inc.). Mean values with standard deviations and 95% confidence intervals (CI), mean difference between groups (95% CI), and median values with minimum–maximum were presented for patient demographic data. Comparative analyses were used to describe differences in patient demographic data and KE strength for patients who had and had not achieved PASS.

To determine how the Q_Nm/kg_ and Q_LSI_ were able to predict the achievement of PASS, odds ratios (OR) with 95% CIs were calculated. Receiver Operating Characteristic curves were constructed to determine the individual predictive capacity for the achievement of PASS of the Q_Nm/kg_ and that of the Q_LSI_, respectively, for which their accuracy was presented using the area under the curve (AUC). Accuracy was determined by the general rules of thumb of Hosmer et al. [10] defined as *outstanding* discrimination (AUC ≥ 0.9), *excellent* discrimination (0.8 ≤ AUC < 0.9), *acceptable* discrimination (0.7 ≤ AUC < 0.8), *poor* discrimination, that is, not much better than a coin flip (0.5 < AUC < 0.7), and *no* discrimination, that is, the same value as a coin flip (AUC = 0.5). Cut‐off values at which sensitivity and specificity were maximized for the achievement of PASS were determined using Youden's *J* index that ranges from −1 to 1 and has a zero value when a test gives the same proportion of positive results for groups with and without PASS. A value of 1 indicates that there are no false positives or false negatives [33].

To account for potential factors influencing KE strength, the analyses regarding reference values were stratified by sex and three age groups (16–19 years; 20–30 years; 31+ years). The analyses regarding the predictive capacity of KE strength were stratified by sex and two age groups (16–24 years; 25+ years) to allow for an adequate number of cases for the Receiver Operating Characteristic curves. Other factors influencing the choices for age ranges were generalized ages for skeletal maturity [27], as well as typical life‐stage transitions in Sweden, such as decreased level of physical activity due to studies, work commitments and parenthood.

All analyses were considered statistically significant if *p* < 0.05.

## RESULTS

**Figure 1:**
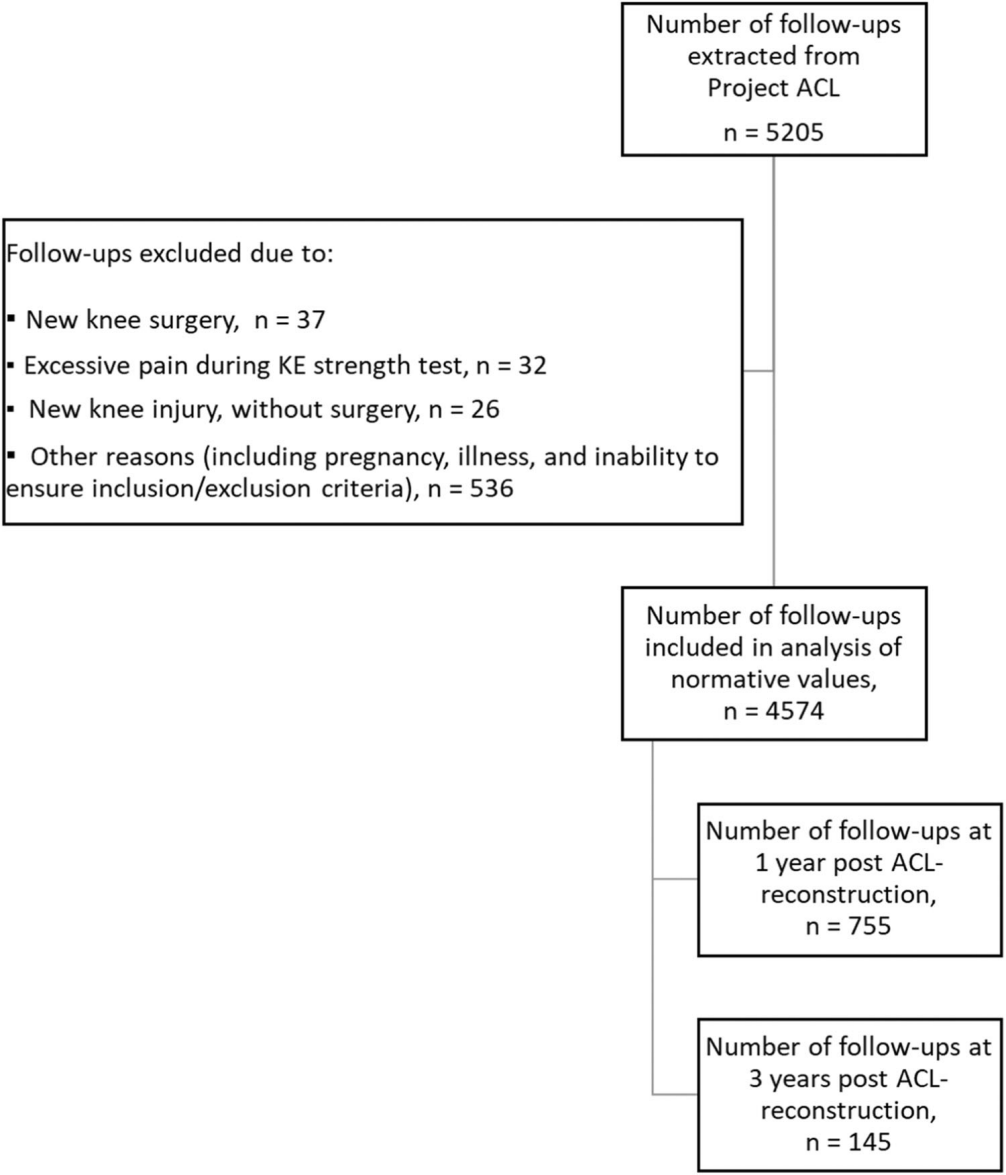
Flow chart of inclusion and exclusion criteria. ACL, anterior cruciate ligament; KE, knee extensor.

Data from a total of 5205 follow‐ups, from 1490 unique patients, were extracted from the registry. After exclusion (Figure 1), demographic data for patients from 4574 (88%) follow‐ups were obtained (Tables 1 and 2 for the 1‐ and 3‐year follow‐ up. Appendices 1–8 for groups related to the reference values). Reasons for exclusion of follow‐ups were new knee surgery (*n* = 37), excessive pain when performing the KE strength test (*n* = 32), and new knee injury without surgery (*n* = 26). Other reasons include pregnancy, illness, and the inability to ensure inclusion/exclusion criteria (*n* = 536).

**Table 1:** Demographic data for patients who achieved PASS compared to patients who did not achieve PASS, stratified by age groups and sex, at 1 year following ACL‐reconstruction.

	Male, 16–24 years			*p* Value	Mean difference between groups (95% CI)	Male, 25+ years			*p* Value	Mean difference between groups (95% CI)
	Total (*n* = 162)	PASS: NO (*n* = 109)	PASS: YES (n = 53)			Total (*n* = 212)	PASS: NO (*n* = 171)	PASS: YES (*n* = 41)		
Age [years]	20.3 (2.8)	20.7 (2.8)	19.4 (2.5)	0.0064*	1.32	34.4 (8.3)	34.3 (8.3)	34.8 (8.3)	0.69	−0.521
	20.1 (16; 25)	21 (16; 25)	19 (16; 24.6)		(0.41; 2.19)	31.4 (25; 59.7)	31.1 (25; 59.7)	33.6 (25.2; 56.8)		(−3.245; 2.455)
Height [cm]	181 (6.6)	182 (6.4)	181 (7.0)	0.68	0.495	180 (6.3)	180 (6.1)	181 (7.2)	0.82	−0.279
	182 (162; 200)	182 (164: 200)	181 (162; 195)		(−1.703; 2.697)	180 (162; 194)	180 (162; 192)	180 (165; 194)		(−2.484; 1.828)
Weight [kg]	79 (10.5)	79 (10.3)	78 (10.9)	0.54	1.14	82 (9.2)	82 (9.2)	81 (9.2)	0.75	0.506
	78 (53; 114)	78 (53; 108)	76 (58; 114)		(−2.24; 4.69)	82 (56; 114)	82 (57; 114)	80 (56; 98)		(−2.576; 3.576)
BMI [kg/m^2^]	24.0 (2.8)	24.1 (2.9)	23.8 (2.8)	0.63	0.237	25.1 (2.3)	25.1 (2.4)	24.9 (1.8)	0.49	0.273
	23.6 (17.5; 33.7)	23.5 (17.5; 32.9)	23.7 (18.1; 33.7)		(−0.690; 1.220)	25.0 (18.0; 33.2)	25.1 (18.0; 33.2)	24.7 (20.6; 29)		(−0.501; 1.031)
Preinjury activity level^a^										
1–5	6 (3.7%)	5 (4.6%)	1 (1.9%)			42 (20.0%)	32 (19.6%)	9 (21.9%)		
6	6 (3.7%)	5 (4.6%)	1 (1.9%)			26 (12.4%)	21 (12.4%)	5 (12.2%)		
7	23 (14.3%)	14 (13.0%)	9 (17.0%)			53 (25.2%)	45 (26.6%)	8 (19.5%)		
8	36 (22.4%)	21 (19.4%)	15 (28.3%)			23 (11.0%)	17 (10.1%)	6 (14.6%)		
9	53 (32.9%)	37 (34.3%)	16 (30.2%)			50 (23.8%)	42 (24.9%)	8 (19.5%)		
10	37 (23.0%)	26 (24.1%)	11 (20.8%)	0.84		16 (7.6%)	11 (6.5%)	5 (12.2%)	0.91	
Missing	1	1	0			2	2	0		
Graft										
HT	115 (71.0%)	76 (69.7%)	39 (73.6%)			181 (85.8%)	142 (83.5%)	39 (95.1%)		
PT	44 (27.2%)	32 (29.4%)	12 (22.6%)			23 (10.9%)	21 (12.4%)	2 (4.9%)		
Quadriceps	0	0	0			3 (1.4%)	3 (1.8%)	0		
Allograft	0	0	0			1 (0.5%)	1 (0.6%)	0		
Other	3 (1.9%)	1 (0.9%)	2 (3.8%)	0.33		3 (1.4%)	3 (1.8%)	0	0.42	
Missing	0	0	0			1	1	0		
Side of graft harvest										
Contralateral	5 (3.1%)	3 (2.8%)	2 (3.8%)	1.00	−1.0 (−8.4; 6.4)	9 (4.3%)	7 (4.1%)	2 (4.9%)	1.00	−0.8 (−9.5; 8.0)
Missing	0	0	0			1	1	0		

	Female, 16–24 years					Female, 25+ years				
	Total (*n* = 205)	PASS: NO (*n* = 154)	PASS: YES (*n* = 51)	*p* Value	Mean difference between groups (95% CI)	Total (*n* = 176)	PASS: NO (*n* = 152)	PASS: YES (*n* = 24)	*p* Value	Mean difference between groups (95% CI)
Age [years]	19.8 (2.6)	19.9 (2.7)	19.3 (2.6)	0.17	0.603	37.6 (9.5)	37.4 (9.7)	38.8 (8.3)	0.50	−1.37
	19.3 (16; 25)	19.5 (16; 25)	18.7 (16; 24.9)		(−0.232; 1.452)	36.9 (25; 63.8)	36.9 (25; 63.8)	38.4 (27.7; 56.9)		(−5.45; 2.88)
Height [cm]	169 (6.1)	169 (6.0)	169 (6.3)	0.61	−0.463	168 (6.0)	168 (6.1)	168 (5.1)	0.96	0.090
	169 (153; 186)	169 (153; 185)	170 (155; 186)		(−2.436; 1.526)	168 (156; 188)	168 (156; 188)	169 (157; 178)		(−2.421; 2.684)
Weight [kg]	66 (8.6)	66 (8.2)	66 (9.5)	0.83	0.373	67 (10.3)	67 (10.4)	68 (9.6)	0.83	−0.430
	65 (45; 109) n = 204	65 (47; 109)	64 (45; 89)		(−2.297; 3.317)	65 (49; 103)	65 (49; 103)	65 (53; 90)		(−4.591; 4.062)
		n = 153								
BMI [kg/m^2^]	23.1 (2.7)	23.2 (2.7)	22.9 (2.8)	0.46	0.324	23.7 (3.0)	23.7 (3.0)	23.9 (3.1)	0.73	−0.208
	22.7 (17.3; 38.2)	22.9 (17.3; 38.2)	22.5 (17.3; 29.1)		(−0.488; 1.197)	23.1 (18.4; 34)	23.1 (18.4; 34)	23.2 (19.9; 33.1)		(−1.407; 1.119)
	*n* = 204	*n* = 153								
Preinjury activity level^a^										
1–5	16 (8.0%)	14 (9.2%)	2 (3.9%)			71 (40.7%)	61 (40.4%)	10 (41.7%)		
6	7 (3.4%)	6 (3.9%)	1 (2.0%)			36 (20.6%)	29 (19.2%)	7 (29.2%)		
7	26 (12.7%)	19 (12.4%)	7 (13.7%)			29 (16.6%)	25 (16.6%)	4 (16.7%)		
8	68 (33.3%)	52 (34.0%)	16 (31.4%)			21 (12.0%)	19 (12.6%)	2 (8.3%)		
9	63 (30.9%)	45 (29.4%)	18 (35.3%)			10 (5.7%)	10 (6.6%)	0		
10	24 (11.8%)	17 (11.1%)	7 (13.7%)	0.098		8 (4.6%)	7 (4.6%)	1 (4.2%)	0.82	
Missing	1	1	0			1	1	0		
Graft										
HT	143 (69.8%)	105 (68.2%)	38 (74.5%)			154 (88.5%)	130 (86.7%)	24 (100.0%)		
PT	55 (26.8%)	44 (28.6%)	11 (21.6%)			14 (8.0%)	14 (9.3%)	0		
Quadriceps	3 (1.5%)	2 (1.3%)	1 (2.0%)			4 (2.3%)	4 (2.7%)	0		
Allograft	2 (1.0%)	1 (0.6%)	1 (2.0%)			2 (1.1%)	2 (1.3%)	0		
Other	2 (1.0%)	2 (1.3%)	0	0.67		0	0	0	0.31	
Missing	0	0	0			2	2	0		
Side of graft harvest										
Contralateral	13 (6.3%)	9 (5.8%)	4 (7.8%)	0.82	−2.0 (−11.6; 7.6)	13 (7.5%)	12 (8.0%)	1 (4.2%)	0.88	3.8 (−7.7; 15.3)
Missing	0	0	0			2	2	0		

*Note*: For categorical variables, *n* (%) is presented. For continuous variables Mean (SD)/Median (Min; Max) is presented. For comparison between groups Fisher's Exact test (lowest 1‐sided *p* value multiplied by 2) was used for dichotomous variables, the Mantel–Haenszel *χ* ^2^ test was used for ordered categorical variables, *χ* ^2^ test was used for nonordered categorical variables and the Fisher's nonparametric permutation test was used for continuous variables. The CI for dichotomous variables is the unconditional exact confidence limits. If no exact limits can be computed the asymptotic Wald confidence limits with continuity correction are calculated instead. The CI for the mean difference between groups is based on Fisher's nonparametric permutation test.

Abbreviations: ACL, anterior cruciate ligament; BMI, body mass index; cm, centimetres; CI, confidence interval; HT, hamstring tendon; kg, kilogram; m, metre; *n*, number; N/A, not applicable; PASS, patient‐acceptable symptom state; PT, patellar tendon.

^a^ Tegner Activity Scale.

* Significant value.

**Table 2:** Demographic data for patients who achieved PASS compared to patients who did not achieve PASS, stratified by age groups and sex, at 3 years following ACL‐reconstruction.

	Male, 16–24 years			*p* Value	Mean difference between groups (95% CI)	Male, 25+ years			*p* Value	Mean difference between groups (95% CI)
	Total (*n* = 16)	PASS: NO (*n* = 10)	PASS: YES (*n* = 6)			Total (*n* = 53)	PASS: NO (*n* = 35)	PASS: YES (*n* = 18)		
Age (years)	20.5 (2.6)	20.9 (2.5)	20.0 (2.8)	0.51	0.912	36.6 (9.0)	37.2 (9.8)	35.2 (7.4)	0.45	2.01
	19.6 (17.3; 24.8)	21.2 (17.3; 24.8)	18.6 (17.6; 24.5)		(−1.995; 3.855)	33.9 (25.8; 57.5)	34.9 (25.8; 57.5)	33.7 (27.2; 54.1)		(−3.21; 7.35)
Height (cm)	179 (7.1)	178 (7.8) 180.5 (164; 188)	181 (6.0)	0.50	−2.67	181 (6.1)	181 (6.8)	180 (4.6)	0.80	0.470
	182 (164; 188)		182 (173; 188)		(−10.75; 5.00)	180 (165; 195)	180 (165; 195)	180 (175; 190)		(−3.125; 4.000)
Weight (kg)	79 (15.0)	82 (13.7)	76 (17.5)	0.46	6.10	80 (9.0)	82 (10.5)	78 (4.9)	0.25	3.01
	77 (57; 108)	77 (58; 108)	71.5 (57; 105)		(−10.67; 23.50)	80 (56; 107)	83 (56; 107)	79.5 (71; 90)		(−2.18; 8.20)
BMI (kg/m^2^)	24.7 (4.1)	25.7 (4.0)	22.9 (4.1)	0.20	2.78	24.6 (2.3)	24.9 (2.7)	24.1 (1.4)	0.26	0.755
	24.4 (19.0; 32.9)	25.2 (21.6; 32.9)	22.1 (19.0; 29.7)		(−1.42; 7.37)	24.5 (20.5; 30.7)	24.7 (20.5; 30.7)	24.4 (21.3; 26.1)		(−0.575; 2.074)
Preinjury activity level^a^										
1–5	1 (6.3%)	1 (10.0%)	0			17 (32.1%)	12 (34.3%)	5 (27.9%)		
6	0	0	0			7 (13.2%)	5 (14.3%)	2 (11.1%)		
7	4 (25.0%)	3 (30.0%)	1 (16.7%)			16 (30.2%)	12 (34.3%)	4 (22.2%)		
8	2 (12.5%)	1 (10.0%)	1 (16.7%)			6 (11.3%)	1 (2.9%)	5 (27.8%)		
9	7 (43.8%)	4 (40.0%)	3 (50.0%)			6 (11.3%)	4 (11.4%)	2 (11.1%)		
10	2 (12.5%)	1 (10.0%)	1 (16.7%)	0.34		1 (1.9%)	1 (2.9%)	0	0.47	
Missing	0	0	0			0	0	0		
Graft										
HT	16 (100.0%)	10 (100.0%)	6 (100.0%)			50 (96.2%)	33 (97.1%)	17 (94.4%)		
PT	0	0	0			2 (3.8%)	1 (2.9%)	1 (5.6%)		
Quadriceps	0	0	0			0	0	0		
Allograft	0	0	0			0	0	0		
Other	0	0	0	N/A		0	0	0	0.64	
Missing	0	0	0			1	1	0		
Side of graft harvest										
Contralateral	0	0	0	N/A		1 (1.9%)	0	1 (5.6%)	0.69	−5.6 (−27.3; 5.6)
Missing	0	0	0			1	1	0		

	Female, 16–24 years					Female, 25+ years				
	Total (*n* = 34)	PASS: NO (*n* = 21)	PASS: YES (*n* = 13)	*p* Value	Mean difference between groups (95% CI)	Total (*n* = 42)	PASS: NO (*n* = 26)	PASS: YES (*n* = 16)	*p* Value	Mean difference between groups (95% CI)
Age (years)	20.6 (2.8) 20.5 (16.1; 24.9)	20.6 (2.8) 19.9 (16.1; 24.9)	20.6 (3.1) 21.1 (16.5; 24.4)	0.97	0.034 (−2.007; 2.077)	36.7 (9.2) 35.5 (25.9; 63.8)	36.8 (10.3) 32.3 (25.9; 63.8)	36.4 (7.3) 36.6 (25.9; 47.7)	0.90	0.422 (−5.306; 6.362)
Height (cm)	167 (6.2)	167 (6.1)	168 (6.5)	0.54	−1.46	167 (6.2) 167.5 (157; 188)	167 (6.7) 167.5 (159; 188)	166 (5.5)	0.63	1.01
	168 (156; 179)	168 (156; 179)	170 (159; 178)		(−6.00; 3.12)			168 (157; 173)		(−2.91; 5.11)
Weight (kg)	64 (8.5)	65 (9.2)	64 (7.5)	0.80	0.784	65 (10.2)	65 (10.1)	65 (10.6)	0.96	0.221
	64.5 (45; 82)	65 (45; 80)	64 (52; 82)		(−5.200; 7.000)	62.5 (49; 98)	62 (53; 98)	63.5 (49; 89)		(−6.300; 7.091)
BMI (kg/m^2^)	22.9 (2.5)	23.2 (2.8)	22.5 (1.9)	0.44	0.674	23.2 (2.5)	23.1 (2.3)	23.3 (2.9)	0.80	−0.207
	22.5 (18.5; 28.3)	23.1 (18.5; 28.3)	22.2 (20.3; 27.1)		(−1.077; 2.473)	22.9 (19.4; 31.9)	22.8 (19.5; 29.8)	23 (19.4; 31.9)		(−1.811; 1.455)
Preinjury activity level^a^										
1–5	6 (17.6%)	3 (14.3%)	3 (23.1%)			14 (33.3%)	7 (26.8%)	7 (43.8%)		
6	1 (2.9%)	1 (4.8%)	0			10 (23.8%)	5 (19.2%)	5 (31.3%)		
7	3 (8.8%)	3 (14.3%)	0			5 (11.9%)	4 (15.4%)	1 (6.3%)		
8	12 (35.3%)	6 (28.6%)	6 (46.2%)			8 (19.0%)	7 (26.9%)	1 (6.3%)		
9	7 (20.6%)	4 (19.0%)	3 (23.1%)			4 (9.5%)	3 (11.5%)	1 (6.3%)		
10	5 (14.7%)	4 (19.0%)	1 (7.7%)	0.65		1 (2.4%)	0	1 (6.3%)	0.20	
Missing	0	0	0			0	0	0		
Graft										
HT	27 (81.8%)	14 (70.0%)	13 (100.0%)			39 (95.1%)	24 (96.0%)	15 (93.8%)		
PT	4 (12.1%)	4 (20.0%)	0			2 (4.9%)	1 (4.0%)	1 (6.3%)		
Quadriceps	1 (3.0%)	1 (5.0%)	0			0	0	0		
Allograft	1 (3.0%)	1 (5.0%)	0			0	0	0		
Other	0	0	0	0.19		0	0	0	0.74	
Missing	1	1	0			1	1	0		
Side of graft harvest										
Contralateral	2 (6.1%)	2 (10.0%)	0	0.72	10.0 (−15.7; 32.0)	3 (7.3%)	3 (12.0%)	0	0.43	12.0 (−9.0; 31.9)
Missing	1	1	0			1	1	0		

*Note*: For categorical variables, *n* (%) is presented. For continuous variables Mean (SD)/Median (Min; Max) is presented. For comparison between groups Fisher's Exact test (lowest 1‐sided *p* value multiplied by 2) was used for dichotomous variables, the Mantel–Haenszel *χ* ^2^ test was used for ordered categorical variables, *χ* ^2^ test was used for nonordered categorical variables and the Fisher's nonparametric permutation test was used for continuous variables. The CI for dichotomous variables is the unconditional exact confidence limits. If no exact limits can be computed the asymptotic Wald confidence limits with continuity correction are calculated instead. The CI for the mean difference between groups is based on Fisher's nonparametric permutation test.

Abbreviations: ACL, anterior cruciate ligament; BMI, body mass index; cm, centimetres; CI, confidence interval; HT, hamstring tendon; kg, kilogram; m, metre; *n*, number; N/A, not applicable; PASS, patient‐acceptable symptom state; PT, patellar tendon.

^a^ Tegner Activity Scale.

### Predictive capacity of KE strength

The results from the follow‐ups of 755 and 145 patients (females≈ 51% and 52%, Tables 1 and 2) were used in the 1‐ and 3‐year follow‐up analyses. PASS was achieved by 169 (22%) and 53 (37%) patients at the 1‐ and 3‐year follow‐ups. Male and female patients (across all ages) achieving PASS at the 1‐year follow‐up were 2.9 years (95% CI: 0.55; 5.14) and 3.1 (0.24; 5.92) younger at their index ACLR compared with patients who did not achieve PASS (*p* = 0.012; 0.032). Higher preinjury physical activity level was reported among female patients (across all ages) achieving PASS at the 1‐year follow‐up compared with patients who did not achieve PASS (*p* = 0.024). Among male patients 16–24 years old, those achieving PASS at the 1‐year follow‐up were 1.3 years (0.41; 2.19) younger compared with patients who did not achieve PASS (*p* = 0.0064). There were no other between‐group differences in patient demographics at any of the follow‐ups.

#### Absolute muscle strength

At the 1‐year follow‐up, male and female patients (both age groups) with ≥2.6 Q_Nm/kg_ and ≥2.1 Q_Nm/kg_, respectively, in the injured limb had significantly higher odds (OR = 2.09–5.12) of achieving PASS. Poor discrimination was revealed by the AUC for cut‐off values for the Q_Nm/kg_.

At the 3‐year follow‐up, female patients 16–24 years old with ≥2.6 Q_Nm/kg_ in the injured limb had significantly higher odds (OR = 36.44) of achieving PASS. The AUC revealed acceptable discrimination between patients who achieved PASS compared with patients who did not achieve PASS for male and female patients 16–24 years old with ≥3.4 Q_Nm/kg_ and ≥2.6 Q_Nm/kg_ in the injured limb, and for male patients 16–24 years old with ≥3.2 Q_Nm/kg_ in the noninjured limb (AUC = 0.700–0.780).

No other significant results were found in any of the age groups. Cut‐off values, sensitivity, specificity, Youden's *J* Index, and the AUC for the Q_Nm/kg_ are presented in Tables 3 and 4.

**Table 3:** Cut‐off values of peak isokinetic knee extension strength, ORs and receiver operating characteristic statistics, 1 year post‐ACL reconstruction.

Male	16–24 years						25+ years					
	Cut‐off	OR (95% CI)	Youden's *J* Index	Specificity	Sensitivity	AUC (95% CI)	Cut‐off	OR (95% CI)	Youden's *J* Index	Specificity	Sensitivity	AUC (95% CI)
Q_Nm/kg_ injured	2.7	2.09^a^ (1.01–4.34)	0.19	0.30	0.89	0.590 (0.499–0.681)	2.6	3.97^a^(1.72–9.17)	0.38	0.43	0.95	0.683 (0.604–0.762)
Q_Nm/kg_noninjured	2.9	1.09 (0.52–2.30)	0.13	0.35	0.77	0.483 (0.391–0.576)	2.6	1.17 (0.65–2.10)	0.22	0.27	0.95	0.573 (0.488–0.659)
Q_LSI_	94.7	1.04^a^(1.01–1.07)	0.26	0.52	0.74	0.618 (0.530–0.707)	91.6	1.05^a^(1.02–1.09)	0.28	0.50	0.78	0.669 (0.584–0.755)
Female												
Q_Nm/kg_injured	2.3	5.12^a^(2.10–12.44)	0.22	0.30	0.92	0.652 (0.566–0.739)	2.1	3.51^a^ (1.26–9.79)	0.34	0.51	0.83	0.658 (0.553–0.763)
Q_Nm/kg_noninjured	3.0	2.23 (0.97–5.12)	0.16	0.77	0.39	0.587 (0.494–0.680)	2.3	1.21 (0.39–3.80)	0.20	0.36	0.83	0.513 (0.408–0.619)
Q_LSI_	103.0	1.05^a^(1.01–1.08)	0.24	0.90	0.33	0.622 (0.530–0.714)	96.6	1.07^a^(1.02–1.12)	0.35	0.72	0.63	0.698 (0.593–0.804)

Abbreviations: ACL, anterior cruciate ligament; AUC, area under the curve; CI, confidence interval; kg, kilogram; OR, odds ratio; Q_LSI_, relative knee extensor strength; Q_Nm/kg_, absolute knee extensor strength.

^a^ Significant value.

**Table 4:** Cut‐off values of peak isokinetic knee extension strength, ORs and receiver operating characteristic statistics, 3 years post‐ACL reconstruction.

Male	16–24 years						25+ years					
	Cut‐off	OR (95% CI)	Youden's *J* Index	Specificity	Sensitivity	AUC (95% CI)	Cut‐off	OR (95% CI)	Youden's *J* Index	Specificity	Sensitivity	AUC (95% CI)
Q_Nm/kg_injured	3.4	3.28 (0.42–25.39)	0.73	0.90	0.83	0.767(0.455–1.000)^a^	3.4	2.22 (0.47–10.40)	0.17	1.00	0.17	0.533 (0.357–0.708)
Q_Nm/kg_noninjured	3.2	3.84 (0.36–41.25)	0.53	0.70	0.83	0.700(0.411–0.989)^a^	2.7	1.52 (0.39–5.96)	0.16	0.38	0.78	0.505 (0.341–0.669)
Q_LSI_	95.5	1.01 (0.92–1.11)	0.30	0.30	1.00	0.500 (0.197–0.803)	103.0	1.05 (0.97–1.14)	0.41	0.86	0.56	0.640 (0.463–0.817)
Female												
Q_Nm/kg_injured	2.6	36.44(1.95–682.71)^b^	0.53	0.76	0.77	0.780(0.619–0.942)^a^	2.4	3.07 (0.64–14.69)	0.40	0.71	0.69	0.641 (0.449–0.832)
Q_Nm/kg_ noninjured	2.5	3.92 (0.37–41.99)	0.29	0.52	0.77	0.630 (0.426–0.834)	2.6	4.76 (0.87–25.98)	0.44	0.87	0.56	0.669 (0.484–0.855)
Q_LSI_	102.0	1.15(1.02–1.29)^b^	0.48	0.71	0.77	0.755(0.577–0.932)^a^	101.0	1.00 (0.94–1.07)	0.28	0.65	0.63	0.524 (0.334–0.714)

Abbreviations: ACL, anterior cruciate ligament; AUC, area under the curve; CI, confidence interval; kg, kilogram; OR, odds ratio; Q_LSI_, relative knee extensor strength; Q_Nm/kg_, absolute knee extensor strength.

^a^ Acceptable discrimination.

^b^ Significant value.

#### LSI

At the 1‐year follow‐up, male and female patients (both age groups) with ≥91.6% Q_LSI_ and ≥96.6% Q_LSI_ had significantly higher odds (OR = 1.04–1.07) of achieving PASS. Poor discrimination was revealed by the AUC for cut‐off values for the Q_LSI._

At the 3‐year follow‐up, female patients 16–24 years old with ≥102%Q_LSI_ had significantly higher odds (OR = 1.15) of achieving PASS. The AUC revealed acceptable discrimination between patients who achieved PASS compared with patients who did not achieve PASS for female patients 16–24 years old with ≥102%Q_LSI_ (AUC = 0.755).

No other significant results were found in any of the age groups. Cut‐off values, sensitivity, specificity, Youden's *J* Index, and the AUC for the Q_LSI_ are presented in Tables 3 and 4.

#### Reference values for isokinetic KE strength

Among males, reference values for the Q_Nm/kg_ in the injured limb, noninjured limb, and the Q_LSI_ ranged for the three age groups between 1.95 and 3.26 Nm/kg, 2.66 and 3.43 Nm/kg and 70.6 and 102.5%, respectively, at follow‐ups between 10 weeks and 5 years after ACLR. Reference values for males, stratified by age groups, are presented in detail in Table 5.

**Table 5:** Reference values for knee extensor strength 10 weeks to 5 years after ACL reconstruction, among male patients, stratified by age groups.

	m2	m4	m8	m12	m18	m24	m36	m48	m60
16–19 years									
*n* of patients	*n* = 78	*n* = 100	*n* = 82	*n* = 77	*n* = 31	*n* = 27	*n* = 8	*n* = 1	*n* = 1
Q_Nm/kg_ injured	2.28 (0.53) 2.30 (1.12; 3.33) *n* = 75	2.58 (0.56) 2.59 (1.49; 3.80) *n* = 98	2.94 (0.56) 2.95 (1.46; 4.75) *n* = 81	3.06 (0.47) 3.09 (1.92; 4.64) *n* = 76	3.12 (0.40) 3.20 (2.04; 3.78) *n* = 31	3.12 (0.47) 3.22 (1.99; 4.01) *n* = 24	3.26 (0.60)3.31 (2.31; 4.27) *n* = 8	3.20	3.14
Q_Nm/kg_ noninjured	2.96 (0.46) 3.03 (1.06; 4.35) *n* = 75	3.02 (0.45) 3.05 (1.67; 4.29) *n* = 98	3.13 (0.46) 3.12 (2.15; 5.08) *n* = 81	3.11 (0.47) 3.08 (2.22; 4.77) *n* = 76	3.12 (0.40) 3.03 (2.21; 4.19) *n* = 31	3.15 (0.41) 3.18 (2.18; 3.99) *n* = 24	3.21 (0.48) 3.35 (2.29; 3.84) *n* = 8	3.29	3.43
Q_LSI_	75.9 (16.2) 77.3 (39.4; 121.1) *n* = 77	85.5 (16.0) 88.4 (43.1; 129.4) *n* = 100	94.1 (12.7) 94.7 (57.8; 135.3) *n* = 82	98.9 (12.6) 98 (73.5; 154.8) *n *= 77	100.5 (9.8) 100.9 (66.7; 114.8) *n* = 31	99.7 (13.2) 96.8 (60.8; 121.3) *n* = 27	101.4 (8.2) 99.1 (94.6; 120.8) *n* = 8	97.5	91.5

20–30 years									
*n* of patients	*n* = 209	*n* = 240	*n* = 244	*n* = 186	*n* = 103	*n* = 76	*n* = 27	*n* = 14	*n* = 12
Q_Nm/kg_ injured	2.09 (0.53) 2.10 (0.60; 3.43) *n* = 207	2.43 (0.55) 2.45 (0.79; 3.97) *n *= 235	2.74 (0.50) 2.75 (1.38; 4.22) *n* = 234	2.86 (0.46) 2.90 (1.43; 4.27) *n* = 178	2.98 (0.41) 2.97 (2.2; 3.9) *n* = 100	3.03 (0.52) 3.02 (1.87; 4.33) *n* = 73	2.89 (0.46) 2.77 (2.03; 4.16) *n *= 27	3.03 (0.38) 2.95 (2.54; 3.80) *n* = 14	2.87 (0.39) 2.96 (2.00; 3.45) *n* = 11
Q_Nm/kg_ noninjured	2.89 (0.42) 2.92 (0.93; 3.98) *n* = 207	3.02 (0.42) 3.05 (1.86; 4.16) *n* = 235	3.06 (0.43) 3.06 (1.92; 4.20) *n* = 234	3.08 (0.56) 3.04 (1.75; 7.6) *n* = 178	3.06 (0.40) 3.05 (1.96; 3.99) *n* = 100	3.03 (0.44) 3.03 (1.95; 4.54) *n* = 73	2.94 (0.51) 3.06 (1.41; 3.93) *n *= 27	2.97 (0.31) 2.90 (2.55; 3.50) *n* = 14	2.89 (0.39) 2.85 (2.46; 3.69) *n* = 11
Q_LSI_	71.9 (15.5) 74.4 (24; 107.3) *n* = 209	80.4 (14.7) 83.2 (28; 120.5) *n* = 240	89.8 (12.6) 92.5 (49.9; 122.9) *n* = 244	93.6 (12.8) 94.4 (36.6; 159.7) *n* = 186	97.8 (7.9) 98.5 (74.6; 121.3) *n* = 103	100.0 (8.7) 100.8 (71.3; 118.1) *n* = 76	98.0 (10.3) 101.6 (70.8; 115.1) *n* = 27	102.0 (7.0) 99.7 (91.8; 117.8) *n* = 14	100.3 (14.3) 100.6 (61.5; 115.6) *n* = 12

31+ years									
*n* of patients	*n* = 130	*n* = 151	*n* = 143	*n* = 111	*n* = 90	*n* = 64	*n* = 34	*n* = 13	*n* = 13
Q_Nm/kg_ injured	1.95 (0.59) 2.00 (0.64; 4.73) *n* = 129	2.16 (0.55) 2.26 (0.53; 3.38) *n* = 149	2.43 (0.53) 2.46 (0.76; 4.06) *n* = 142	2.56 (0.49) 2.60 (1.12; 3.53) *n* = 109	2.65 (0.41) 2.65 (1.63; 3.52) *n* = 88	2.74 (0.37) 2.67 (1.71; 3.49) *n* = 59	2.77 (0.33) 2.71 (2.15; 3.41) *n* = 33	2.69 (0.49) 2.55 (2.04; 3.84) *n* = 13	2.86 (0.36) 2.90 (2.21; 3.47) *n* = 13
Q_Nm/kg_ noninjured	2.74 (0.46) 2.71 (1.86; 4.73) *n* = 129	2.77 (0.41) 2.74 (0.93; 3.77) *n* = 149	2.81 (0.40) 2.77 (1.85; 4.38) *n* = 142	2.83 (0.39) 2.84 (1.53; 3.87) *n* = 109	2.79 (0.41) 2.78 (1.68; 3.64) *n* = 88	2.82 (0.38) 2.76 (1.83; 3.61) *n* = 59	2.72 (0.50) 2.78 (0.27; 3.20) *n* = 33	2.66 (0.41) 2.66 (2.06; 3.37) *n* = 13	2.79 (0.26) 2.81 (2.44; 3.32) *n* = 13
Q_LSI_	70.6 (16.7) 71.8 (23.3; 111.1) *n* = 130	77.7 (15.8) 80.3 (24.9; 110) *n* = 151	86.5 (14.6) 88.2 (29.8; 113.2) *n* = 143	90.8 (12.8) 91.4 (45.1; 117.4) *n* = 111	95.0 (10.4) 96.6 (63.5; 121) *n* = 90	97.3 (10.7) 97.9 (67.8; 127) *n *= 64	99.2 (8.0) 100.4 (77.2; 110.1) *n* = 34	101.1 (10.1) 101.8 (81; 116.9) *n* = 13	102.5 (8.8) 102.4 (81.8; 114.5) *n* = 13

*Note*: Values are presented as ‘mean (SD) median (min–max)’.

Abbreviations: ACL, anterior cruciate ligament; m, months; m2, 10 weeks follow‐up; *n*, number; Q_LSI_, relative knee extensor strength; Q_Nm/kg_, absolute knee extensor strength.

Among females, reference values for the Q_Nm/kg_ in the injured limb, noninjured limb, and the Q_LSI_ ranged for the three age groups between 1.47 and 2.66 Nm/kg, 2.05 and 2.72 Nm/kg and 66 and 108.2%, respectively, at follow‐ups between 10 weeks and 5 years after ACLR. Reference values for females, stratified by age groups, are presented in detail in Table 6.

**Table 6:** Reference values for knee extensor strength 10 weeks to 5 years after ACL reconstruction, among female patients, stratified by age groups.

	m2	m4	m8	m12	m18	m24	m36	m48	m60
16–19 years									
*n* of patients	*n* = 107	*n* = 139	*n* = 141	*n* = 119	*n* = 63	*n* = 30	*n* = 16	*n* = 9	*n* = 4
Q_Nm/kg_ injured	1.79 (0.49) 1.80 (0.76; 3.16) *n* = 105	2.11 (0.44) 2.13 (0.84; 3.34) *n* = 138	2.41 (0.41) 2.41 (1.25; 3.34) *n* = 138	2.57 (0.41) 2.57 (1.40; 3.44) *n* = 116	2.66 (0.46) 2.63 (1.00; 3.44) *n* = 60	2.63 (0.46) 2.54 (1.59; 3.46) *n* = 29	2.55 (0.35) 2.57 (1.73; 3.14) *n* = 16	2.61 (0.35) 2.47 (2.24; 3.21) *n* = 8	2.59 (0.28) 2.49 (2.35; 3.00) *n* = 4
Q_Nm/kg_ noninjured	2.60 (0.36) 2.55 (1.89; 3.75) *n* = 105	2.68 (0.39) 2.64 (1.59; 4.06) *n* = 138	2.69 (0.39) 2.70 (1.07; 3.68) *n* = 138	2.72 (0.41) 2.73 (1.60; 3.64) *n* = 116	2.72 (0.38) 2.64 (1.94; 3.51) *n* = 60	2.57 (0.46) 2.57 (1.50; 3.45) *n* = 29	2.53 (0.30) 2.55 (2.02; 3.16) *n* = 16	2.47 (0.29) 2.41 (2.13; 2.96) *n* = 8	2.39 (0.25) 2.39 (2.09; 2.69) *n* = 4
Q_LSI_	68.9 (16.1) 69.5 (32.2; 103.9) *n* = 107	79.4 (14.3) 81.2 (34; 108.1) *n* = 139	89.6 (12.4) 91 (51.4; 125.0) *n* = 141	94.9 (11.2) 95.2 (52.6; 116.3) *n* = 119	97.8 (11.3) 99 (46.6; 121.8) *n* = 63	102.4 (7.3) 101.9 (87.1; 119.9) *n* = 30	101.2 (9.7) 102.4 (73.8; 113.5) *n* = 16	104.8 (6.4) 105 (94; 113.4) *n* = 9	108.2 (4.7) 108.9 (102.5; 112.5) *n* = 4

20–30 years									
*n* of patients	*n* = 194	*n* = 209	*n* = 202	*n* = 150	*n* = 87	*n* = 59	*n* = 36	*n* = 18	*n* = 18
Q_Nm/kg_ injured	1.74 (0.46) 1.75 (0.48; 3.03) *n* = 191	1.98 (0.46) 1.97 (0.55; 3.16) *n* = 206	2.28 (0.46) 2.27 (0.85; 3.71) *n* = 198	2.43 (0.45) 2.45 (0.91; 3.92) *n* = 147	2.50 (0.51) 2.46 (1.40; 4.07) *n* = 84	2.47 (0.47) 2.40 (1.46; 3.55) *n* = 55	2.49 (0.34) 2.47 (1.63; 3.01) *n* = 35	2.49 (0.64) 2.40 (1.01; 3.74) *n* = 17	2.55 (0.56) 2.43 (1.72; 3.74) *n* = 18
Q_Nm/kg_ noninjured	2.55 (0.37) 2.54 (1.54; 3.56) *n* = 191	2.58 (0.39) 2.57 (0.86; 3.77) *n* = 206	2.63 (0.39) 2.65 (1.58; 3.73) *n* = 198	2.67 (0.38) 2.67 (1.66; 3.58) *n* = 147	2.62 (0.43) 2.63 (1.78; 3.62) *n* = 84	2.55 (0.39) 2.54 (1.64; 3.54) *n* = 55	2.51 (0.42) 2.48 (1.48; 3.48) *n* = 35	2.45 (0.52) 2.38 (1.65; 3.59) *n* = 17	2.53 (0.46) 2.51 (1.93; 3.83) *n* = 18
Q_LSI_	68.3 (16.2) 72.1 (21.8; 102.4) *n* = 194	77.1 (16.4) 78.7 (24.3; 118.5) *n* = 209	86.8 (13.3) 88.0 (41.6; 119.3) *n* = 202	90.9 (11.9) 92.1 (42.7; 130.6) *n* = 150	95.3 (11.2) 95.7 (59.3; 130.2) *n* = 87	96.4 (9.7) 95.7 (72.4; 130.3) *n* = 59	98.2 (8.5) 98.6 (81.5; 115.2) *n* = 36	102.6 (22.0) 98.8 (55.5; 160.6) *n* = 18	100.7 (13.1) 99.7 (82.9; 140.6) *n* = 18

31+ years									
*n* of patients	*n* = 122	*n* = 143	*n* = 146	*n* = 112	*n* = 89	*n* = 50	*n* = 24	*n* = 12	*n* = 10
Q_Nm/kg_ injured	1.47 (0.41) 1.45 (0.58; 2.55) *n* = 121	1.73 (0.42) 1.72 (0.71; 2.64) *n* = 142	1.98 (0.38) 1.96 (0.97; 3.12) *n* = 145	2.04 (0.41) 2.07 (0.67; 3.06) *n* = 109	2.16 (0.41) 2.22 (1.28; 3.12) *n* = 86	2.18 (0.37) 2.16 (1.40; 3.18) *n* = 48	2.31 (0.47) 2.28 (1.53; 3.31) *n* = 23	2.02 (0.31) 1.98 (1.52; 2.51) *n* = 12	2.11 (0.37) 2.03 (1.60; 2.89) *n* = 10
Q_Nm/kg_ noninjured	2.23 (0.37) 2.24 (1.31; 3.03) *n* = 121	2.31 (0.36) 2.28 (1.43; 3.62) *n* = 142	2.31 (0.34) 2.30 (1.38; 3.43) *n* = 145	2.26 (0.34) 2.28 (1.25; 3.15) *n* = 109	2.30 (0.37) 2.28 (1.28; 3.27) *n* = 86	2.24 (0.35) 2.24 (1.21; 3.15) *n* = 48	2.30 (0.36) 2.31 (1.62; 3.00) *n* = 23	2.05 (0.36) 2.06 (1.32; 2.49) *n* = 12	2.16 (0.30) 2.07 (1.70; 2.63) *n* = 10
Q_LSI_	66.0 (14.7) 66.2 (24.9; 101.5) *n* = 122	75.0 (14.5) 76.4 (30.1; 105.5) *n* = 143	85.6 (13.0) 86.8 (31.3; 114) *n* = 146	90.2 (12.4) 92.2 (36.4; 119) *n* = 112	94.0 (10.1) 95.1 (65.4; 115.7) *n* = 88	97.0 (11.7) 97.7 (63.4; 124.6) *n* = 50	100.0 (9.7) 99 (85.5; 120.1) *n *= 24	99.5 (9.4) 97.9 (85.6; 116.7) *n* = 12	97.2 (6.9) 96.2 (86.0; 109.8) *n* = 10

*Note*: Values are presented as ‘mean (SD) median (min–max)’.

Abbreviations: ACL, anterior cruciate ligament; m, months; m2, 10 weeks follow‐up; *n*, number; Q_LSI_, relative knee extensor strength; Q_Nm/kg_, absolute knee extensor strength.

## DISCUSSION

The main findings in this study were that patients of both sexes and age groups who achieved the cut‐off values for absolute KE strength at the 1‐year follow‐up had two to five times the odds of achieving PASS. At the 3‐year follow‐up, absolute KE strength showed acceptable predictive capacity for achieving PASS, among male and female patients 16–24 years old. Moreover, the reference values presented may facilitate interpretation of KE strength assessments, targeted to six subgroups, during the first five years after an ACLR.

Although the ORs for achieving PASS by the Q_LSI_ are statistically significant, they are not considered clinically relevant. The results of our study are in agreement with the results reported by Pietrosimone et al. [18] regarding the positive association between higher absolute KE strength and achieving PASS. However, we were unable to fully confirm previous findings of a positive association between the LSI in KE strength and self‐reported knee function [5, 11, 18, 22]. The divergent results may be explained by the use of different PROMs, for example, the KOOS and the International Knee Documentation Committee index for assessing self‐reported knee function, making it difficult to compare results between studies. In addition, differences in sample sizes may result in divergent findings. For example, our cohort of males and female patients 16–24 years old was about five times higher than that in Ithurburn et al. [11] including patients 13–25 years old. Interestingly, they found a higher proportion of patients (67%) who had a patellar tendon graft in the group who had <85% Q_LSI_ at ≈20 months after ACLR, whereas we had a cohort with the inverse proportion and no difference in graft harvesting site (hamstring tendon ≈70%; *p* = 0.33–0.67) between patients 16–24 years old who had achieved PASS compared to those who had not, at 1 year after ACLR. Ultimately, the use of the LSI has a limitation: it does not account for lower KE strength in the contralateral limb, which our results indicate. As a result, the use of the LSI may overestimate KE strength after ACLR [17, 19, 31]. Despite this, current guidelines recommend the use of the LSI for assessing KE strength during the rehabilitation process [13, 29, 30].

With the limitations of the LSI in mind, the cut‐off values of the Q_Nm/kg_ at which PASS is reached in the present study are worthy of further discussion. Pietrosimone et al. [18] reported a cut‐off value of ≥3.1 Q_Nm/kg_ for isometric KE strength that had an acceptable accuracy (AUC = 0.76) for identifying patients (mean age = 21.7 years) achieving PASS at an average of 3 years after ACLR. We assessed isokinetic KE strength for which a lower cut‐off value for absolute KE strength is expected, with respect to the force‐velocity curve. For males and females 16–24 years old (mean age ≈20.5 years), we found a cut‐off value of ≥3.4 Q_Nm/kg_ and ≥2.6 Q_Nm/kg_ for identifying patients achieving PASS, with acceptable accuracy (AUC = 0.767– 0.780) at the 3‐year follow‐up. Pietrosimone et al. [18] presented the cut‐off value for males and females combined (mean age = 21.7 years), which may explain the deviation found in our results as male sex has been reported to influence absolute KE strength [9, 14]. Furthermore, a difference in graft harvesting site between the two studies may have influenced the slightly higher than expected isokinetic compared to isometric KE strength. The hamstring tendon was used for ACLR among 81.8%–100% of the patients in our study, in contrast to 38.5% in Pietrosimone et al. [18], where the patellar tendon was more common (58.3%).

Pietrosimone et al. [18] reported that quadriceps strength normalized to body weight was a stronger predictor of subjective knee function compared with the LSI 3 years after ACLR. We, therefore, hypothesized that absolute isokinetic KE strength normalized to body weight would have a better capacity to discriminate between patients who had achieved and not achieved PASS in the KOOS subscales compared with the Q_LSI_. Our hypothesis was partially corroborated as all cut‐off values showed significantly higher ORs for achieving PASS at the 1‐year follow‐up, but arguably, only the cut‐off values for Q_Nm/kg_ being clinically relevant. In addition, the AUC showed acceptable discriminative capacity for Q_Nm/kg_ in the injured limb for males and females 16–24 years old, and for the Q_LSI_ for females in the same age group, at the 3‐year follow‐up. The low discriminative capacity (AUC = 0.483–0.698) and Youden's *J* Index found for most cut‐off values in the current study indicates that these should not be used as solitary measures to identify patients that consider themselves as feeling well. The low AUC can be explained by several factors, mainly the use of PASS in this study, that are discussed below. Despite the low AUC, cut‐off values for Q_Nm/kg_ may pose as a valuable piece of the puzzle in the evaluation of knee function at 1 year after ACLR, with respect to the clinically relevant ORs.

There were some limitations that were considered before any conclusions were drawn from this study. One limitation was the inconsistent ways of reporting concomitant injuries potentially influencing KE strength and/or subjective knee function [9], which means that the eligibility of two different patients with the same concomitant injury may have been inconsistently determined. Our data consisted of reports from the patients at each follow‐up, via supplementary questions when answering the PROMs, and from the examiner and the patients' responsible physical therapist, also at each follow‐up.

Furthermore, the KOOS has been reported to entail some limitations, specifically regarding ceiling effects and low content validity for the Pain and ADL subscales [29], as well as the lack of questions regarding knee‐related instability. However, the KOOS is one of the most commonly used PROMs [6] for assessing patients after an ACLR, and it was therefore chosen.

A further limitation is our use of the PASS. In the current study, only 37% of patients achieved PASS at the 3‐year follow‐up, compared with the findings of Muller et al. [14], where 89% achieved PASS between 1 and 5 years after ACLR (mean follow‐up = 3.4 years). Muller et al. [14] used the question ‘Taking into account all the activity you have during your daily life, your level of pain, and also your activity limitations and participation restrictions, do you consider the current state of your knee satisfactory?’ to determine the achievement of PASS, while patients in the current study who may have been in an acceptable symptom state but only surpassed the threshold of four of five subscales were defined as not achieving PASS. The lower proportion of patients who achieved PASS in the current study may also be explained by the age (mean = 26.1 years at ACLR) [14] of the cohort used to establish the PASS thresholds. The younger age group in our study included patients who were adolescent at the time of ACLR and may be less likely to achieve PASS compared to young adults [27]. Conversely, the patients in our older age group were, on average, roughly 9 years older than the patients in the study by Muller et al. [14] and similar to the age of the cohort in a recently published paper presenting lower PASS thresholds for long‐term evaluation after ACLR [28]. This new data facilitates the use of more appropriate PASS thresholds for patients with various characteristics in future research.

While grouping of hamstring and patellar tendon grafts for KE strength assessment offers a comprehensive view, it is important to acknowledge potential limitations. Variations in surgical techniques, graft harvesting site, patient characteristics and rehabilitation factors could introduce confounding factors that may impact the comparability of outcomes between the two graft types, especially at early follow‐ups after ACLR [20].

The main strength of this study, however, is the large cohort who have undergone ACLR and the use of common and reliable test methods. We included 1490 unique patients, with 58,982 patients distributed at the nine different follow‐ups. However, our results, which are based on nine different follow‐ups, include partly the same cohort of patients from different follow‐ups. To describe changes over time, future studies should aim to present longitudinal reference values for absolute, as well as relative KE strength during the first 5 years following an ACLR, including follow‐ups at several time points and for the same cohort of patients.

To account for factors influencing KE strength, we presented reference values by three age groups each among males and females, for isokinetic KE strength during the first 5 years after an ACLR, for clinicians to use in daily practice.

## CONCLUSION

At 1 year after ACLR, patients of both sexes and age groups reaching cut‐off values for absolute KE strength had two to five times the odds, that were clinically relevant, to achieve PASS. Acceptable discriminative capacity was found for the absolute KE strength among male and female patients 16–24 years old, at 3 years after ACLR.

## AUTHOR CONTRIBUTIONS

All the authors contributed to planning the project. Farshad Ashnai and Susanne Beischer drafted the manuscript. All the authors critically revised the manuscript and approved the final version of the manuscript.

## CONFLICT OF INTEREST STATEMENT

The authors declare no conflicts of interest.

## ETHICS STATEMENT

The data have been coded and none of the included patients could be identified during analyses. The study was approved by the Regional Ethical Review Board in Gothenburg and the Swedish Ethical Review Authority (registration numbers: 265‐13, T023‐17, 2020‐02501). All the included patients provided written informed consent.

## Supporting information

Supporting information. [Correction added on 9 July 2025, after first online publication: The supplemental tables that appeared as Appendices are now online as Supporting Information.]

## Data Availability

The data set used and/or analysed is available from the corresponding author on request.
