# Supplementary material for: Higher isokinetic quadriceps peak force is associated with a patient‐acceptable symptom‐state 1 and 3 years after ACL reconstruction
Source: Knee Surg Sports Traumatol Arthrosc. 2024 Nov 20;33(8):2847–65. doi: 10.1002/ksa.12541 (PMC12310089; doi:10.1002/ksa.12541)
Supplement: Supplementary file 1 — Supporting information. [Correction added on 9 July 2025, after first online publication: The supplemental tables that appeared as Appendices are now online as Supporting Information.] [file KSA-33-2847-s001.docx]

# Appendices

| Appendix 1: Demographic data for males, all ages. | | | | | | | | | |
| --- | --- | --- | --- | --- | --- | --- | --- | --- | --- |
|  | m2 | m4 | m8 | m12 | m18 | m24 | m36 | m48 | m60 |
| n of patients | n=417 | n=491 | n=469 | n=374 | n=224 | n=167 | n=69 | n=28 | n=26 |
| Age [years] | 28.7 (9.6) 26.7  (16; 59.7) n=417 | 28.5 (9.7) 26.4  (16; 59.7) n=491 | 28.7 (9.7) 26.4  (16; 59.7) n=469 | 28.3 (9.5) 26.5  (16; 59.7) n=374 | 30.5 (10.4) 27.7  (16.6; 59.7) n=224 | 30.2 (9.9) 27.6  (16.4; 59.7) n=167 | 32.8 (10.5) 30.1  (17.3; 57.5) n=69 | 33.0 (8.5) 30  (18.6; 53.5) n=28 | 31.5 (7.7) 30.7  (18.6; 50.1) n=26 |
| Height [cm] | 182 (6.5) 182  (162; 201) n=407 | 181 (6.6) 181  (163; 209) n=472 | 182 (6.5) 182  (162; 209) n=441 | 181 (6.6) 180.5  (162; 200) n=346 | 181 (6.4) 180.5  (162; 198) n=204 | 180 (6.5) 180  (162; 199) n=145 | 181 (6.4) 181  (165; 195) n=65 | 179 (4.8) 178.5  (168; 190) n=28 | 179 (6.6) 179  (165; 195) n=23 |
| Weight [kg] | 83 (11.1) 82  (54; 121) n=412 | 83 (12.0) 82  (53; 174) n=482 | 83 (10.3) 82  (52; 130) n=457 | 81 (10.0) 80  (55; 117) n=363 | 82 (10.6) 80  (55; 120) n=219 | 82 (10.2) 80  (54; 122.4) n=156 | 81 (11.1) 79.2  (56; 115) n=68 | 80 (7.8) 80  (65; 95.5) n=28 | 80 (10.4) 81  (55; 97) n=25 |
| BMI [kg/m^2^] | 25.2 (3.0) 24.9  (17.5; 38.0) n=407 | 25.3 (3.4) 24.9  (16.7; 59.5) n=472 | 25.1 (2.8) 24.8  (16.4; 38.8) n=441 | 24.9 (2.7) 24.7  (17.9; 37.6) n=346 | 24.9 (2.8) 24.8  (19.7; 35.9) n=204 | 25.0 (2.7) 24.8  (18.2; 37.0) n=145 | 24.7 (3.0) 24.5  (19.7; 34.7) n=65 | 25.0 (2.3) 24.8  (20.8; 31.1) n=28 | 24.7 (2.9) 24.9  (19.7; 30.0) n=23 |
| Pre-injury activity level^1^ | |  |  |  |  |  |  |  |  |
| 1 | 1 (0.2%) | 1 (0.2%) | 3 (0.6%) | 3 (0.8%) | 3 (1.4%) | 1 (0.6%) | 1 (1.4%) | 1 (3.6%) | 1 (3.8%) |
| 2 | 10 (2.4%) | 6 (1.2%) | 4 (0.9%) | 5 (1.3%) | 4 (1.8%) | 3 (1.8%) | 1 (1.4%) |  | 1 (3.8%) |
| 3 | 6 (1.4%) | 9 (1.8%) | 6 (1.3%) | 4 (1.1%) | 4 (1.8%) | 3 (1.8%) | 2 (2.9%) | 1 (3.6%) |  |
| 4 | 16 (3.8%) | 19 (3.9%) | 21 (4.5%) | 17 (4.6%) | 11 (5.0%) | 11 (6.6%) | 7 (10.1%) | 5 (17.9%) | 2 (7.7%) |
| 5 | 20 (4.8%) | 26 (5.3%) | 18 (3.9%) | 19 (5.1%) | 11 (5.0%) | 11 (6.6%) | 7 (10.1%) | 1 (3.6%) | 2 (7.7%) |
| 6 | 36 (8.7%) | 40 (8.2%) | 39 (8.4%) | 32 (8.6%) | 25 (11.3%) | 14 (8.4%) | 7 (10.1%) | 4 (14.3%) |  |
| 7 | 94 (22.6%) | 93 (19.1%) | 90 (19.3%) | 76 (20.5%) | 51 (23.1%) | 44 (26.5%) | 20 (29.0%) | 10 (35.7%) | 11 (42.3%) |
| 8 | 60 (14.4%) | 78 (16.0%) | 80 (17.1%) | 59 (15.9%) | 29 (13.1%) | 23 (13.9%) | 8 (11.6%) |  | 3 (11.5%) |
| 9 | 119 (28.6%) | 135 (27.7%) | 133 (28.5%) | 103 (27.8%) | 55 (24.9%) | 38 (22.9%) | 13 (18.8%) | 5 (17.9%) | 5 (19.2%) |
| 10 | 54 (13.0%) | 80 (16.4%) | 73 (15.6%) | 53 (14.3%) | 28 (12.7%) | 18 (10.8%) | 3 (4.3%) | 1 (3.6%) | 1 (3.8%) |
| Graft |  |  |  |  |  |  |  |  |  |
| HT | 327 (79.8%) | 383 (79.0%) | 376 (81.0%) | 296 (79.4%) | 191 (85.7%) | 147 (88.6%) | 66 (97.1%) | 27 (96.4%) | 24 (96.0%) |
| PT | 71 (17.3%) | 89 (18.4%) | 77 (16.6%) | 67 (18.0%) | 27 (12.1%) | 17 (10.2%) | 2 (2.9%) | 1 (3.6%) | 1 (4.0%) |
| Quadriceps | 3 (0.7%) | 3 (0.6%) | 3 (0.6%) | 3 (0.8%) | 3 (1.3%) | 1 (0.6%) |  |  |  |
| Allograft | 1 (0.2%) | 2 (0.4%) | 2 (0.4%) | 1 (0.3%) | 1 (0.4%) |  |  |  |  |
| Other | 8 (2.0%) | 8 (1.6%) | 6 (1.3%) | 6 (1.6%) | 1 (0.4%) | 1 (0.6%) |  |  |  |
| Side of graft harvest | |  |  |  |  |  |  |  |  |
| Contralateral | 17 (4.1%) | 23 (4.7%) | 21 (4.5%) | 14 (3.8%) | 8 (3.6%) | 4 (2.4%) | 1 (1.5%) |  |  |
| For categorical variables n (%) is presented. For continuous variables Mean (SD) / Median (Min; Max) / n= is presented. BMI= Body Mass Index; cm= centimetres; kg= kilogram; m= months; m2= 10 weeks follow-up; m^2^= metres squared; n= number; ^1^= Tegner Activity Scale. | | | | | | | | | |

| Appendix 2: Demographic data for males 16 – 19 years | | | | | | | | | |
| --- | --- | --- | --- | --- | --- | --- | --- | --- | --- |
|  | m2 | m4 | m8 | m12 | m18 | m24 | m36 | m48 | m60 |
| n of patients | n=78 | n=100 | n=82 | n=77 | n=31 | n=27 | n=8 | n=1 | n=1 |
| Age [years] | 17.9 (1.1) 18.0  (16.0; 19.9) n=78 | 17.9 (1.1) 17.8  (16.0; 20) n=100 | 17.9 (1.1) 18.0  (16.0; 20) n=82 | 17.7 (1.1) 17.7  (16.0; 20) n=77 | 18.0 (1.0) 17.8  (16.6; 20) n=31 | 18.1 (0.9) 18.2  (16.4; 19.5) n=27 | 18.3 (0.6) 18.5  (17.3; 18.9) n=8 | 18.6 | 18.6 |
| Height [cm] | 181 (6.5) 180  (163; 201) n=76 | 181 (6.7) 181  (163; 201) n=97 | 182 (6.1) 182  (164; 195) n=78 | 181 (7.2) 181  (162; 200) n=71 | 182 (7.0) 183  (165; 198) n=27 | 183 (8.2) 182  (165; 199) n=22 | 181 (8.1) 184  (165; 188) n=7 | 185 | 18 |
| Weight [kg] | 78 (11.6) 75.4  (58; 116) n=76 | 78 (11.4) 75.5  (57; 130) n=98 | 79 (11.1) 77  (59; 130) n=81 | 78 (9.8) 77  (58; 117) n=76 | 79 (11.5) 78  (61; 113) n=31 | 77 (9.4) 78  (59; 103) n=24 | 76 (9.6) 79.5  (60; 86) n=8 | 84.0 | 86.0 |
| BMI [kg/m^2^] | 23.9 (3.5) 23.3  (17.5; 34.8) n=76 | 23.8 (3.2) 23.2  (18.2; 38.8) n=97 | 23.9 (3.3) 23.3  (17.6; 38.8) n=78 | 23.6 (2.7) 23  (17.9; 34.6) n=71 | 23.7 (3.0) 22.7  (19.9; 33.4) n=27 | 23.0 (2.3) 22.8  (18.2; 27.1) n=22 | 23.1 (1.6) 22.8  (20.5; 24.8) n=7 | 24.5 | 25.1 |
| Pre-injury activity level^1^ | |  |  |  |  |  |  |  |  |
| 1 | 1 (1.3%) |  | 1 (1.2%) | 1 (1.3%) | 1 (3.2%) |  |  |  |  |
| 4 | 2 (2.6%) | 3 (3.0%) | 2 (2.4%) | 2 (2.6%) | 1 (3.2%) | 1 (3.7%) |  |  |  |
| 5 |  |  | 1 (1.2%) | 1 (1.3%) |  |  |  |  |  |
| 7 | 9 (11.5%) | 10 (10.0%) | 9 (11.0%) | 10 (13.0%) | 5 (16.1%) | 5 (18.5%) | 2 (25.0%) | 1 (100.0%) | 1 (100.0%) |
| 8 | 15 (19.2%) | 22 (22.0%) | 21 (25.6%) | 17 (22.1%) | 6 (19.4%) | 5 (18.5%) | 1 (12.5%) |  |  |
| 9 | 24 (30.8%) | 30 (30.0%) | 23 (28.0%) | 27 (35.1%) | 12 (38.7%) | 11 (40.7%) | 3 (37.5%) |  |  |
| 10 | 27 (34.6%) | 35 (35.0%) | 25 (30.5%) | 19 (24.7%) | 6 (19.4%) | 5 (18.5%) | 2 (25.0%) |  |  |
| Graft |  |  |  |  |  |  |  |  |  |
| HT | 52 (66.7%) | 66 (67.3%) | 60 (74.1%) | 55 (71.4%) | 26 (83.9%) | 25 (92.6%) | 8 (100.0%) | 1 (100.0%) | 1 (100.0%) |
| PT | 24 (30.8%) | 29 (29.6%) | 18 (22.2%) | 19 (24.7%) | 5 (16.1%) | 2 (7.4%) |  |  |  |
| Other | 2 (2.6%) | 3 (3.1%) | 3 (3.7%) | 3 (3.9%) |  |  |  |  |  |
| Side of graft harvest | |  |  |  |  |  |  |  |  |
| Contralateral | 5 (6.4%) | 5 (5.1%) | 5 (6.2%) | 4 (5.2%) | 3 (9.7%) | 1 (3.7%) |  |  |  |
| For categorical variables n (%) is presented. For continuous variables Mean (SD) / Median (Min; Max) / n= is presented. BMI= Body Mass Index; cm= centimetres; kg= kilogram; m= months; m2= 10 weeks follow-up; m^2^= metres squared; n= number; ^1^= Tegner Activity Scale. | | | | | | | | | |

| Appendix 3: Demographic data for males 20 – 30 years | | | | | | | | | |
| --- | --- | --- | --- | --- | --- | --- | --- | --- | --- |
|  | m2 | m4 | m8 | m12 | m18 | m24 | m36 | m48 | m60 |
| n of patients | n=209 | n=240 | n=244 | n=186 | n=103 | n=76 | n=27 | n=14 | n=12 |
| Age [years] | 25.4 (3.0) 25.5  (20.0; 30.8) n=209 | 25.3 (3.0) 25.3  (20.0; 30.8) n=240 | 25.2 (2.9) 25.1  (20.0; 30.8) n=244 | 25.4 (3.0) 25.6  (20.0; 31) n=186 | 25.0 (3.0) 25.0  (20.0; 30.7) n=103 | 25.6 (2.9) 26.3  (20.0; 30.6) n=76 | 26.7 (2.9) 27.2  (20.2; 30.1) n=27 | 27.6 (2.2) 28.3  (22.8; 30.1)  n=14 | 25.9 (3.0) 25.9  (20.9; 29.8) n=12 |
| Height [cm] | 182 (6.6) 183  (162; 198) n=205 | 182 (6.7) 183  (165; 209) n=228 | 182 (6.8) 183  (162; 209) n=226 | 181 (6.7) 181  (162; 197) n=170 | 180 (6.9) 181  (162; 194) n=92 | 180 (6.6) 180  (162; 197) n=65 | 180 (7.1) 181  (165; 191) n=27 | 179 (5.4) 178  (168; 190) n=14 | 178 (7.3) 180  (165; 187) n=11 |
| Weight [kg] | 84 (10.3) 83  (54; 120) n=207 | 84 (10.8) 83  (53; 115) n=235 | 83 (10.2) 83  (52; 120) n=234 | 82 (10.1) 82  (55; 110) n=178 | 80 (11.2) 80  (55; 120) n=100 | 83 (11.0) 84  (54; 115) n=73 | 82 (14.4) 79.3  (56; 115) n=27 | 79 (9.0) 78.5  (65; 94) n=14 | 77 (13.4) 75  (55; 97) n=11 |
| BMI [kg/m^2^] | 25.3 (2.7) 24.9  (17.8; 35.4) n=205 | 25.2 (2.9) 24.9  (16.7; 34) n=228 | 25.1 (2.7) 24.7  (16.4; 34.9) n=226 | 25.0 (2.7) 24.8  (18.3; 33.2) n=170 | 24.6 (2.9) 24.4  (19.7; 35.4) n=92 | 25.4 (2.8) 25.4  (19.4; 34.7) n=65 | 25.1 (3.8) 24.5  (19.7; 34.7) n=27 | 24.8 (2.8) 24.6  (20.8; 31.1) n=14 | 24.3 (3.7) 24.3  (19.7; 30) n=11 |
| Pre-injury activity level^1^ | |  |  |  |  |  |  |  |  |
| 1 |  | 1 (0.4%) | 1 (0.4%) | 1 (0.5%) | 1 (1.0%) | 1 (1.3%) |  |  |  |
| 2 | 4 (1.9%) | 2 (0.8%) | 3 (1.2%) | 2 (1.1%) | 2 (2.0%) | 2 (2.6%) | 1 (3.7%) |  | 1 (8.3%) |
| 3 | 2 (1.0%) | 2 (0.8%) | 2 (0.8%) | 2 (1.1%) | 1 (1.0%) | 1 (1.3%) |  |  |  |
| 4 | 4 (1.9%) | 6 (2.5%) | 6 (2.5%) | 7 (3.8%) | 4 (3.9%) | 6 (7.9%) | 4 (14.8%) | 4 (28.6%) |  |
| 5 | 9 (4.3%) | 13 (5.5%) | 8 (3.3%) | 6 (3.3%) | 4 (3.9%) | 5 (6.6%) | 3 (11.1%) |  | 1 (8.3%) |
| 6 | 8 (3.8%) | 8 (3.4%) | 10 (4.1%) | 11 (6.0%) | 5 (4.9%) | 3 (3.9%) | 2 (7.4%) | 2 (14.3%) |  |
| 7 | 45 (21.5%) | 41 (17.3%) | 34 (14.0%) | 31 (16.9%) | 20 (19.6%) | 14 (18.4%) | 8 (29.6%) | 5 (35.7%) | 5 (41.7%) |
| 8 | 30 (14.4%) | 37 (15.6%) | 43 (17.8%) | 30 (16.4%) | 15 (14.7%) | 14 (18.4%) | 3 (11.1%) |  | 3 (25.0%) |
| 9 | 82 (39.2%) | 89 (37.6%) | 92 (38.0%) | 62 (33.9%) | 32 (31.4%) | 19 (25.0%) | 6 (22.2%) | 3 (21.4%) | 2 (16.7%) |
| 10 | 25 (12.0%) | 38 (16.0%) | 43 (17.8%) | 31 (16.9%) | 18 (17.6%) | 11 (14.5%) |  |  |  |
| Graft |  |  |  |  |  |  |  |  |  |
| HT | 167 (80.3%) | 187 (78.2%) | 192 (79.0%) | 142 (76.3%) | 83 (80.6%) | 64 (85.3%) | 26 (96.3%) | 13 (92.9%) | 11 (91.7%) |
| PT | 33 (15.9%) | 44 (18.4%) | 46 (18.9%) | 40 (21.5%) | 17 (16.5%) | 10 (13.3%) | 1 (3.7%) | 1 (7.1%) | 1 (8.3%) |
| Quadriceps | 3 (1.4%) | 3 (1.3%) | 2 (0.8%) | 2 (1.1%) | 3 (2.9%) | 1 (1.3%) |  |  |  |
| Allograft |  | 1 (0.4%) | 1 (0.4%) |  |  |  |  |  |  |
| Other | 5 (2.4%) | 4 (1.7%) | 2 (0.8%) | 2 (1.1%) |  |  |  |  |  |
| Side of graft harvest | |  |  |  |  |  |  |  |  |
| Contralateral | 8 (3.8%) | 12 (5.0%) | 9 (3.7%) | 7 (3.8%) | 2 (1.9%) | 1 (1.3%) |  |  |  |
| For categorical variables n (%) is presented. For continuous variables Mean (SD) / Median (Min; Max) / n= is presented. BMI= Body Mass Index; cm= centimetres; kg= kilogram; m= months; m2= 10 weeks follow-up; m^2^= metres squared; n= number; ^1^= Tegner Activity Scale. | | | | | | | | | |

| Appendix 4: Demographic data for males 31+ years | | | | | | | | | |
| --- | --- | --- | --- | --- | --- | --- | --- | --- | --- |
|  | m2 | m4 | m8 | m12 | m18 | m24 | m36 | m48 | m60 |
| n of patients | n=130 | n=151 | n=143 | n=111 | n=90 | n=64 | n=34 | n=13 | n=13 |
| Age [years] | 40.5 (7.3) 39.8  (31.1; 59.7) n=130 | 40.5 (7.4) 39.1  (31.0; 59.7) n=151 | 40.9 (7.7) 39.6  (31.1; 59.7) n=143 | 40.4 (7.3) 39.0  (31.1; 59.7) n=111 | 41.1 (7.5) 40.0  (31.1; 59.7) n=90 | 40.7 (6.9) 39.3  (31.1; 59.7) n=64 | 41.2 (8.1) 39.1  (31.3; 57.5) n=34 | 40.0 (7.3) 37.9  (32.5; 53.5) n=13 | 37.7 (5.5) 35.3  (31.5; 50.1) n=13 |
| Height [cm] | 181 (6.3) 180  (164; 195) n=126 | 180 (6.3) 180  (164; 195) n=147 | 180 (6.0) 180  (164; 193) n=137 | 180 (5.8) 179  (165; 193) n=105 | 180 (5.4) 180  (165; 193) n=85 | 180 (5.5) 180  (165; 195) n=58 | 181 (5.4) 180  (172; 195) n=31 | 179 (4.2) 178  (172; 185) n=13 | 180 (6.2) 178  (172; 195) n=11 |
| Weight [kg] | 85 (11.3) 83  (63; 121) n=129 | 85 (13.1) 84  (60; 174) n=149 | 84 (9.4) 83  (65; 120) n=142 | 83 (9.5) 82  (65; 115) n=109 | 84 (9.0) 83  (65; 119) n=88 | 82 (9.3) 80  (64; 122.4) n=59 | 81 (8.1) 79  (68; 96) n=33 | 81 (6.9) 80  (69; 95.5) n=13 | 82 (7.1) 82  (71; 97) n=13 |
| BMI [kg/m^2^] | 26.0 (2.9) 25.6  (20.8; 38.0) n=126 | 26.4 (3.9) 25.7  (20.5; 59.5) n=147 | 25.9 (2.5) 25.5  (20.5; 36.2) n=137 | 25.5 (2.5) 25.1  (20.7; 37.6) n=105 | 25.7 (2.4) 25.1  (21.3; 35.9) n=85 | 25.2 (2.5) 24.8  (20.4; 37.0) n=58 | 24.7 (2.3) 24.8  (20.8; 29.7) n=31 | 25.1 (1.8) 25.1  (22.8; 28.7) n=13 | 25.1 (2.1) 25  (21.3; 29) n=11 |
| Pre-injury activity level^1^ | |  |  |  |  |  |  |  |  |
| 1 |  |  | 1 (0.7%) | 1 (0.9%) | 1 (1.1%) |  | 1 (2.9%) | 1 (7.7%) | 1 (7.7%) |
| 2 | 6 (4.7%) | 4 (2.7%) | 1 (0.7%) | 3 (2.7%) | 2 (2.3%) | 1 (1.6%) |  |  |  |
| 3 | 4 (3.1%) | 7 (4.7%) | 4 (2.8%) | 2 (1.8%) | 3 (3.4%) | 2 (3.2%) | 2 (5.9%) | 1 (7.7%) |  |
| 4 | 10 (7.8%) | 10 (6.7%) | 13 (9.1%) | 8 (7.2%) | 6 (6.8%) | 4 (6.3%) | 3 (8.8%) | 1 (7.7%) | 2 (15.4%) |
| 5 | 11 (8.5%) | 13 (8.7%) | 9 (6.3%) | 12 (10.8%) | 7 (8.0%) | 6 (9.5%) | 4 (11.8%) | 1 (7.7%) | 1 (7.7%) |
| 6 | 28 (21.7%) | 32 (21.3%) | 29 (20.3%) | 21 (18.9%) | 20 (22.7%) | 11 (17.5%) | 5 (14.7%) | 2 (15.4%) |  |
| 7 | 40 (31.0%) | 42 (28.0%) | 47 (32.9%) | 35 (31.5%) | 26 (29.5%) | 25 (39.7%) | 10 (29.4%) | 4 (30.8%) | 5 (38.5%) |
| 8 | 15 (11.6%) | 19 (12.7%) | 16 (11.2%) | 12 (10.8%) | 8 (9.1%) | 4 (6.3%) | 4 (11.8%) |  |  |
| 9 | 13 (10.1%) | 16 (10.7%) | 18 (12.6%) | 14 (12.6%) | 11 (12.5%) | 8 (12.7%) | 4 (11.8%) | 2 (15.4%) | 3 (23.1%) |
| 10 | 2 (1.6%) | 7 (4.7%) | 5 (3.5%) | 3 (2.7%) | 4 (4.5%) | 2 (3.2%) | 1 (2.9%) | 1 (7.7%) | 1 (7.7%) |
| Graft |  |  |  |  |  |  |  |  |  |
| HT | 108 (87.1%) | 130 (87.8%) | 124 (88.6%) | 99 (90.0%) | 82 (92.1%) | 58 (90.6%) | 32 (97.0%) | 13 (100.0%) | 12 (100.0%) |
| PT | 14 (11.3%) | 16 (10.8%) | 13 (9.3%) | 8 (7.3%) | 5 (5.6%) | 5 (7.8%) | 1 (3.0%) |  |  |
| Quadriceps |  |  | 1 (0.7%) | 1 (0.9%) |  |  |  |  |  |
| Allograft | 1 (0.8%) | 1 (0.7%) | 1 (0.7%) | 1 (0.9%) | 1 (1.1%) |  |  |  |  |
| Other | 1 (0.8%) | 1 (0.7%) | 1 (0.7%) | 1 (0.9%) | 1 (1.1%) | 1 (1.6%) |  |  |  |
| Side of graft harvest | |  |  |  |  |  |  |  |  |
| Contralateral | 4 (3.2%) | 6 (4.1%) | 7 (5.0%) | 3 (2.7%) | 3 (3.4%) | 2 (3.1%) | 1 (3.0%) |  |  |
| For categorical variables n (%) is presented. For continuous variables Mean (SD) / Median (Min; Max) / n= is presented. BMI= Body Mass Index; cm= centimetres; kg= kilogram; m= months; m2= 10 weeks follow-up; m^2^= metres squared; n= number; ^1^= Tegner Activity Scale. | | | | | | | | | |

| Appendix 5: Demographic data for females, all ages. | | | | | | | | | |
| --- | --- | --- | --- | --- | --- | --- | --- | --- | --- |
|  | m2 | m4 | m8 | m12 | m18 | m24 | m36 | m48 | m60 |
| n of patients | n=423 | n=491 | n=489 | n=381 | n=239 | n=139 | n=76 | n=39 | n=32 |
| Age [years] | 28.0 (9.9) 25.6  (16.0; 58.0) n=423 | 28.0 (10.7) 24.8  (16.0; 63.8) n=491 | 28.3 (10.9) 24.8  (16.0; 63.8) n=489 | 28.0 (11.2) 24.3  (16.0; 63.8) n=381 | 29.7 (11.3) 26.5  (16.1; 63.8) n=239 | 30.5 (11.9) 27.2  (16.1; 63.8) n=139 | 29.5 (10.7) 26.3  (16.1; 63.8) n=76 | 29.0 (10.4) 26.3  (16.5; 58.4) n=39 | 30.9 (11.8) 26.3  (17.7; 63.8) n=32 |
| Height [cm] | 168 (6.0) 168  (154; 184) n=414 | 168 (6.1) 168  (151; 188) n=477 | 169 (6.0) 169  (150; 186) n=473 | 169 (6.0) 169  (151; 188) n=363 | 169 (6.1) 169  (154; 188) n=224 | 168 (6.3) 169  (153; 188) n=129 | 167 (6.4) 168  (156; 188) n=69 | 168 (6.6) 168  (159; 188) n=36 | 167 (6.4) 168  (158; 188) n=31 |
| Weight [kg] | 67 (9.6) 66  (45; 101) n=417 | 67 (9.5) 66  (46; 108) n=486 | 68 (9.6) 67  (47; 113) n=481 | 68 (9.9) 66  (45; 110) n=372 | 67 (9.9) 66  (49.2; 106) n=231 | 67 (9.1) 65  (47; 99) n=132 | 66 (9.1) 65  (52; 100) n=74 | 66 (9.8) 64.4  (54; 93) n=37 | 64 (9.3) 62.5  (53; 93) n=32 |
| BMI [kg/m^2^] | 23.8 (3.0) 23.3  (17.3; 35.4) n=412 | 23.7 (2.8) 23.4  (17.8; 37.8) n=476 | 23.8 (2.9) 23.4  (17.7; 38.7) n=472 | 23.8 (3.0) 23.4  (17.3; 38.5) n=360 | 23.6 (2.7) 23.1  (17.3; 34.0) n=221 | 23.5 (2.9) 23.1  (17.9; 35.6) n=129 | 23.7 (2.6) 23.3  (19.5; 33.7) n=69 | 23.5 (2.7) 22.8  (19.4; 30.4) n=36 | 23.0 (2.6) 22.2  (18.8; 30.1) n=31 |
| Pre-injury activity level^1^ | |  |  |  |  |  |  |  |  |
| 1 | 5 (1.2%) | 6 (1.2%) | 6 (1.2%) | 2 (0.5%) | 3 (1.3%) | 1 (0.7%) | 2 (2.6%) | 1 (2.6%) | 1 (3.1%) |
| 2 | 15 (3.5%) | 13 (2.7%) | 5 (1.0%) | 12 (3.2%) | 7 (2.9%) | 3 (2.2%) |  |  | 1 (3.1%) |
| 3 | 18 (4.3%) | 23 (4.7%) | 22 (4.5%) | 19 (5.0%) | 15 (6.3%) | 7 (5.0%) | 3 (3.9%) | 2 (5.3%) | 1 (3.1%) |
| 4 | 35 (8.3%) | 33 (6.7%) | 35 (7.2%) | 30 (7.9%) | 25 (10.5%) | 12 (8.6%) | 11 (14.5%) | 3 (7.9%) | 2 (6.3%) |
| 5 | 19 (4.5%) | 26 (5.3%) | 26 (5.3%) | 24 (6.3%) | 12 (5.0%) | 8 (5.8%) | 4 (5.3%) | 2 (5.3%) | 2 (6.3%) |
| 6 | 54 (12.8%) | 55 (11.2%) | 61 (12.5%) | 43 (11.3%) | 40 (16.7%) | 27 (19.4%) | 11 (14.5%) | 6 (15.8%) | 4 (12.5%) |
| 7 | 59 (13.9%) | 69 (14.1%) | 74 (15.2%) | 55 (14.5%) | 32 (13.4%) | 20 (14.4%) | 8 (10.5%) | 4 (10.5%) | 5 (15.6%) |
| 8 | 86 (20.3%) | 108 (22.0%) | 108 (22.1%) | 89 (23.5%) | 45 (18.8%) | 34 (24.5%) | 20 (26.3%) | 11 (28.9%) | 8 (25.0%) |
| 9 | 88 (20.8%) | 103 (21.0%) | 96 (19.7%) | 73 (19.3%) | 45 (18.8%) | 18 (12.9%) | 11 (14.5%) | 5 (13.2%) | 4 (12.5%) |
| 10 | 44 (10.4%) | 54 (11.0%) | 55 (11.3%) | 32 (8.4%) | 15 (6.3%) | 9 (6.5%) | 6 (7.9%) | 4 (10.5%) | 4 (12.5%) |
| Graft |  |  |  |  |  |  |  |  |  |
| HT | 343 (82.3%) | 388 (80.3%) | 387 (80.1%) | 297 (78.4%) | 199 (84.3%) | 126 (91.3%) | 66 (89.2%) | 38 (97.4%) | 29 (90.6%) |
| PT | 58 (13.9%) | 81 (16.8%) | 77 (15.9%) | 69 (18.2%) | 29 (12.3%) | 9 (6.5%) | 6 (8.1%) | 1 (2.6%) | 3 (9.4%) |
| Quadriceps | 8 (1.9%) | 7 (1.4%) | 9 (1.9%) | 7 (1.8%) | 4 (1.7%) | 2 (1.4%) | 1 (1.4%) |  |  |
| Allograft | 1 (0.2%) | 3 (0.6%) | 4 (0.8%) | 4 (1.1%) | 3 (1.3%) | 1 (0.7%) | 1 (1.4%) |  |  |
| Other | 7 (1.7%) | 4 (0.8%) | 6 (1.2%) | 2 (0.5%) | 1 (0.4%) |  |  |  |  |
| Side of graft harvest | |  |  |  |  |  |  |  |  |
| Contralateral | 23 (5.5%) | 30 (6.2%) | 29 (6.0%) | 26 (6.9%) | 14 (5.9%) | 9 (6.5%) | 5 (6.8%) | 3 (7.7%) | 4 (12.5%) |
| For categorical variables n (%) is presented. For continuous variables Mean (SD) / Median (Min; Max) / n= is presented. BMI= Body Mass Index; cm= centimetres; kg= kilogram; m= months; m2= 10 weeks follow-up; m^2^= metres squared; n= number; ^1^= Tegner Activity Scale. | | | | | | | | | |

| Appendix 6: Demographic data for females 16 – 19 years. | | | | | | | | | |
| --- | --- | --- | --- | --- | --- | --- | --- | --- | --- |
|  | m2 | m4 | m8 | m12 | m18 | m24 | m36 | m48 | m60 |
| n of patients | n=107 | n=139 | n=141 | n=119 | n=63 | n=30 | n=16 | n=9 | n=4 |
| Age [years] | 18.0 (1.1) 17.9  (16.0; 20) n=107 | 17.9 (1.1) 17.9  (16.0; 20) n=139 | 17.9 (1.1) 17.9  (16.0; 20) n=141 | 17.8 (1.1) 17.7  (16.0; 19.9) n=119 | 17.9 (1.2) 17.8  (16.1; 19.9) n=63 | 17.6 (1.2) 17.6  (16.1; 19.7) n=30 | 18.0 (1.3) 18.1  (16.1; 19.9) n=16 | 17.6 (1.2) 17.1  (16.5; 19.8) n=9 | 18.4 (1.0) 18.1  (17.7; 19.8) n=4 |
| Height [cm] | 168 (5.8) 168  (154; 179) n=101 | 168 (5.8) 168  (151; 179) n=135 | 168 (6.1) 168  (151; 186) n=135 | 169 (5.6) 169  (151; 186) n=114 | 168 (6.1) 169  (154; 186) n=59 | 168 (7.2) 168.5  (153; 181) n=28 | 168 (6.8) 168.5  (156; 178) n=16 | 171 (7.0) 170  (161; 181) n=8 | 167.8 (3.4) 168.5  (163; 171) n=4 |
| Weight [kg] | 65 (8.6) 64  (45; 87) n=105 | 65 (8.5) 65  (46; 89.5) n=138 | 67 (9.1) 65.1  (47; 104) n=138 | 66 (8.4) 66  (45; 95) n=116 | 66 (8.4) 65  (49.2; 93) n=60 | 66 (8.6) 65  (47; 87.4) n=29 | 67 (10.1) 68.5  (52; 90) n=16 | 70 (10.0) 70  (56; 84) n=8 | 67.8 (9.6) 64  (61; 82) n=4 |
| BMI [kg/m^2^] | 23.0 (2.6) 22.7  (17.3; 30.8) n=101 | 23.0 (2.6) 23  (17.8; 29.9) n=135 | 23.5 (2.9) 23.1  (18.1; 38.7) n=135 | 23.4 (2.6) 23.2  (17.3; 34.5) n=114 | 23.4 (2.7) 22.8  (17.3; 33.0) n=58 | 23.2 (2.6) 22.8  (17.9; 28.9) n=28 | 23.7 (2.9) 23  (19.6; 29.7) n=16 | 23.9 (2.8) 22.9  (21.0; 28.7) n=8 | 24.1 (3.3) 22.6  (22.1; 29.1) n=4 |
| Pre-injury activity level^1^ | |  |  |  |  |  |  |  |  |
| 3 | 2 (1.9%) | 1 (0.7%) | 1 (0.7%) |  | 1 (1.6%) |  |  |  |  |
| 4 | 2 (1.9%) | 2 (1.4%) | 1 (0.7%) | 1 (0.8%) |  |  |  |  |  |
| 5 | 3 (2.8%) | 3 (2.2%) | 3 (2.1%) | 2 (1.7%) | 1 (1.6%) | 2 (6.7%) |  |  |  |
| 6 | 1 (0.9%) | 3 (2.2%) | 3 (2.1%) | 2 (1.7%) | 2 (3.2%) | 1 (3.3%) | 1 (6.3%) |  |  |
| 7 | 11 (10.3%) | 16 (11.5%) | 18 (12.8%) | 12 (10.2%) | 7 (11.1%) | 2 (6.7%) |  |  | 1 (25.0%) |
| 8 | 28 (26.2%) | 42 (30.2%) | 42 (29.8%) | 43 (36.4%) | 19 (30.2%) | 12 (40.0%) | 6 (37.5%) | 5 (62.5%) | 1 (25.0%) |
| 9 | 39 (36.4%) | 46 (33.1%) | 47 (33.3%) | 38 (32.2%) | 22 (34.9%) | 8 (26.7%) | 4 (25.0%) | 1 (12.5%) | 1 (25.0%) |
| 10 | 21 (19.6%) | 26 (18.7%) | 26 (18.4%) | 20 (16.9%) | 11 (17.5%) | 5 (16.7%) | 5 (31.3%) | 2 (25.0%) | 1 (25.0%) |
| Graft |  |  |  |  |  |  |  |  |  |
| HT | 82 (78.1%) | 99 (72.3%) | 104 (74.3%) | 83 (69.7%) | 50 (79.4%) | 27 (90.0%) | 13 (81.3%) | 9 (100.0%) | 3 (75.0%) |
| PT | 21 (20.0%) | 34 (24.8%) | 33 (23.6%) | 32 (26.9%) | 12 (19.0%) | 2 (6.7%) | 2 (12.5%) |  | 1 (25.0%) |
| Quadriceps | 1 (1.0%) | 2 (1.5%) | 1 (0.7%) | 2 (1.7%) |  | 1 (3.3%) | 1 (6.3%) |  |  |
| Allograft |  | 1 (0.7%) | 1 (0.7%) | 1 (0.8%) | 1 (1.6%) |  |  |  |  |
| Other | 1 (1.0%) | 1 (0.7%) | 1 (0.7%) | 1 (0.8%) |  |  |  |  |  |
| Side of graft harvest | |  |  |  |  |  |  |  |  |
| Contralateral | 5 (4.8%) | 8 (5.8%) | 8 (5.7%) | 8 (6.7%) | 3 (4.8%) | 1 (3.3%) | 1 (6.3%) |  | 1 (25.0%) |
| For categorical variables n (%) is presented. For continuous variables Mean (SD) / Median (Min; Max) / n= is presented. BMI= Body Mass Index; cm= centimetres; kg= kilogram; m= months; m2= 10 weeks follow-up; m^2^= metres squared; n= number; ^1^= Tegner Activity Scale. | | | | | | | | | |

| Appendix 7: Demographic data for females 20 – 30 years. | | | | | | | | | |
| --- | --- | --- | --- | --- | --- | --- | --- | --- | --- |
|  | m2 | m4 | m8 | m12 | m18 | m24 | m36 | m48 | m60 |
| n of patients | n=194 | n=209 | n=202 | n=150 | n=87 | n=59 | n=36 | n=18 | n=18 |
| Age [years] | 25.2 (2.9) 24.9  (20.1; 30.6) n=194 | 24.8 (2.9) 24.7  (20.1; 30.8) n=209 | 25.0 (3.1) 24.7  (20.1; 30.8) n=202 | 24.8 (3.2) 24.5  (20.1; 30.8) n=150 | 25.2 (3.0) 25.0  (20.1; 30.8) n=87 | 25.4 (3.1) 24.9  (20.2; 30.6) n=59 | 25.6 (3.1) 25.4  (20.1; 30.6) n=36 | 25.8 (2.7) 26.0  (21.7; 30.5) n=18 | 25.6 (2.7) 26.0  (21.7; 30.2) n=18 |
| Height [cm] | 169 (6.3) 169  (154; 181) n=191 | 169 (6.3) 169  (154; 188) n=200 | 169 (6.2) 169  (150; 185) n=195 | 169 (6.4) 169  (155; 185) n=142 | 168 (6.2) 168  (158; 182) n=82 | 169 (6.2) 169  (158; 180) n=54 | 166 (5.9) 164  (158; 180) n=31 | 166 (5.9) 165.5  (159; 176) n=16 | 165 (5.9) 163.5  (158; 176) n=18 |
| Weight [kg] | 68 (9.6) 67  (48; 100) n=191 | 69 (9.7) 67.5  (48; 108) n=206 | 68 (10.2) 67  (50; 113) n=198 | 69 (10.0) 66  (50; 110) n=147 | 68 (9.3) 67  (52; 93) n=84 | 67 (8.9) 66  (52; 99) n=55 | 66 (7.6) 66  (53; 94) n=35 | 63 (8.3) 60  (54; 88) n=17 | 63 (7.8) 61.5  (55; 87) n=18 |
| BMI [kg/m^2^] | 23.8 (2.9) 23.4  (18.8; 35.4) n=190 | 24.1 (2.9) 23.7  (18.6; 37.8) n=200 | 24.0 (3.1) 23.6  (17.7; 38.5) n=194 | 23.9 (3.0) 23.3  (18.1; 38.5) n=140 | 23.9 (2.8) 23.5  (19.6; 32.3) n=82 | 23.7 (3.2) 23.2  (18.1; 35.5) n=54 | 24.1 (2.5) 23.4  (21.0; 33.7) n=31 | 22.7 (2.7) 22  (19.4; 30.4) n=16 | 23.2 (2.5) 22.4  (20.3; 30.1) n=18 |
| Pre-injury activity level^1^ | |  |  |  |  |  |  |  |  |
| 1 | 1 (0.5%) | 1 (0.5%) | 1 (0.5%) | 1 (0.7%) | 1 (1.1%) |  | 2 (5.6%) | 1 (5.6%) | 1 (5.6%) |
| 2 | 5 (2.6%) | 5 (2.4%) | 2 (1.0%) | 6 (4.0%) | 2 (2.3%) | 1 (1.7%) |  |  | 1 (5.6%) |
| 3 | 5 (2.6%) | 10 (4.8%) | 8 (4.0%) | 8 (5.4%) | 4 (4.6%) | 2 (3.4%) | 2 (5.6%) | 2 (11.1%) | 1 (5.6%) |
| 4 | 12 (6.2%) | 13 (6.3%) | 10 (5.0%) | 9 (6.0%) | 7 (8.0%) | 2 (3.4%) | 4 (11.1%) |  | 1 (5.6%) |
| 5 | 5 (2.6%) | 5 (2.4%) | 7 (3.5%) | 7 (4.7%) | 5 (5.7%) | 3 (5.1%) | 2 (5.6%) | 1 (5.6%) | 1 (5.6%) |
| 6 | 22 (11.3%) | 16 (7.7%) | 21 (10.4%) | 14 (9.4%) | 13 (14.9%) | 9 (15.3%) | 4 (11.1%) | 2 (11.1%) | 1 (5.6%) |
| 7 | 33 (17.0%) | 31 (14.9%) | 31 (15.3%) | 26 (17.4%) | 16 (18.4%) | 13 (22.0%) | 5 (13.9%) | 2 (11.1%) | 2 (11.1%) |
| 8 | 49 (25.3%) | 53 (25.5%) | 55 (27.2%) | 37 (24.8%) | 19 (21.8%) | 18 (30.5%) | 11 (30.6%) | 4 (22.2%) | 5 (27.8%) |
| 9 | 44 (22.7%) | 50 (24.0%) | 43 (21.3%) | 32 (21.5%) | 18 (20.7%) | 8 (13.6%) | 5 (13.9%) | 4 (22.2%) | 3 (16.7%) |
| 10 | 18 (9.3%) | 24 (11.5%) | 24 (11.9%) | 9 (6.0%) | 2 (2.3%) | 3 (5.1%) | 1 (2.8%) | 2 (11.1%) | 2 (11.1%) |
| Graft |  |  |  |  |  |  |  |  |  |
| HT | 158 (81.9%) | 165 (79.3%) | 156 (77.6%) | 114 (76.0%) | 69 (81.2%) | 54 (91.5%) | 30 (85.7%) | 17 (94.4%) | 16 (88.9%) |
| PT | 31 (16.1%) | 39 (18.8%) | 37 (18.4%) | 32 (21.3%) | 13 (15.3%) | 4 (6.8%) | 4 (11.4%) | 1 (5.6%) | 2 (11.1%) |
| Quadriceps | 2 (1.0%) | 1 (0.5%) | 3 (1.5%) | 1 (0.7%) |  |  |  |  |  |
| Allograft |  | 1 (0.5%) | 2 (1.0%) | 2 (1.3%) | 2 (2.4%) | 1 (1.7%) | 1 (2.9%) |  |  |
| Other | 2 (1.0%) | 2 (1.0%) | 3 (1.5%) | 1 (0.7%) | 1 (1.2%) |  |  |  |  |
| Side of graft harvest | |  |  |  |  |  |  |  |  |
| Contralateral | 11 (5.7%) | 11 (5.3%) | 11 (5.5%) | 8 (5.3%) | 4 (4.7%) | 4 (6.8%) | 2 (5.7%) | 2 (11.1%) | 1 (5.6%) |
| For categorical variables n (%) is presented. For continuous variables Mean (SD) / Median (Min; Max) / n= is presented. BMI= Body Mass Index; cm= centimetres; kg= kilogram; m= months; m2= 10 weeks follow-up; m^2^= metres squared; n= number; ^1^= Tegner Activity Scale. | | | | | | | | | |

| Appendix 8: Demographic data for females 31+ years. | | | | | | | | | |
| --- | --- | --- | --- | --- | --- | --- | --- | --- | --- |
|  | m2 | m4 | m8 | m12 | m18 | m24 | m36 | m48 | m60 |
| n of patients | n=122 | n=143 | n=146 | n=112 | n=89 | n=50 | n=24 | n=12 | n=10 |
| Age [years] | 41.3 (6.6) 41.5  (31.3; 58) n=122 | 42.4 (7.3) 42.6  (31.3; 63.8) n=143 | 42.7 (7.2) 42.4  (31.3; 63.8) n=146 | 43.2 (7.4) 42.9  (31.3; 63.8) n=112 | 42.4 (7.3) 42.6  (31.3; 63.8) n=89 | 44.3 (7.7) 44.1  (31.3; 63.8) n=50 | 43.0 (7.1) 42.8  (32.2; 63.8) n=24 | 42.3 (6.9) 41.4  (32.9; 58.4) n=12 | 45.5 (10.1) 42.7  (32.9; 63.8) n=10 |
| Height [cm] | 168 (5.7) 168  (157; 184) n=122 | 168 (6.0) 168  (157; 184) n=142 | 168 (5.7) 169  (156; 184) n=143 | 168 (5.8) 168  (157; 188) n=107 | 168 (6.2) 169  (157; 188) n=83 | 168 (6.1) 169  (159; 188) n=47 | 168 (6.6) 169.5  (157; 188) n=22 | 169 (7.0) 169  (160; 188) n=12 | 170 (7.9) 170  (159; 188) n=9 |
| Weight [kg] | 69 (10.2) 67  (49; 101) n=121 | 68 (9.7) 67  (50; 102) n=142 | 68 (9.2) 67  (50; 100) n=145 | 68 (11.0) 67  (51; 106) n=109 | 68 (11.4) 66  (50; 106) n=87 | 67 (9.7) 65  (51.7; 98) n=48 | 66 (10.8) 64  (52; 100) n=23 | 70 (10.4) 69  (55; 93) n=12 | 65 (12.0) 62 (53; 93) n=10 |
| BMI [kg/m^2^] | 24.3 (3.2) 23.7  (19.6; 33.9) n=121 | 24.0 (2.9) 23.7  (18.4; 33.1) n=141 | 23.8 (2.7) 23.5  (18.4; 32.7) n=143 | 24.1 (3.3) 23.5  (18.5; 37.7) n=106 | 23.4 (2.7) 23  (18.4; 34.0) n=81 | 23.5 (2.8) 23.3  (19.1; 35.6) n=47 | 23.3 (2.3) 23.3  (19.5; 28.3) n=22 | 24.3 (2.6) 23.6  (21.5; 30.1) n=12 | 22.3 (2.4) 22.0  (18.8; 26.3) n=9 |
| Pre-injury activity level^1^ | |  |  |  |  |  |  |  |  |
| 1 | 4 (3.3%) | 5 (3.5%) | 5 (3.4%) | 1 (0.9%) | 2 (2.2%) | 1 (2.0%) |  |  |  |
| 2 | 10 (8.2%) | 8 (5.6%) | 3 (2.1%) | 6 (5.4%) | 5 (5.6%) | 2 (4.0%) |  |  |  |
| 3 | 11 (9.0%) | 12 (8.4%) | 13 (9.0%) | 11 (9.8%) | 10 (11.2%) | 5 (10.0%) | 1 (4.2%) |  |  |
| 4 | 21 (17.2%) | 18 (12.6%) | 24 (16.6%) | 20 (17.9%) | 18 (20.2%) | 10 (20.0%) | 7 (29.2%) | 3 (25.0%) | 1 (10.0%) |
| 5 | 11 (9.0%) | 18 (12.6%) | 16 (11.0%) | 15 (13.4%) | 6 (6.7%) | 3 (6.0%) | 2 (8.3%) | 1 (8.3%) | 1 (10.0%) |
| 6 | 31 (25.4%) | 36 (25.2%) | 37 (25.5%) | 27 (24.1%) | 25 (28.1%) | 17 (34.0%) | 6 (25.0%) | 4 (33.3%) | 3 (30.0%) |
| 7 | 15 (12.3%) | 22 (15.4%) | 25 (17.2%) | 17 (15.2%) | 9 (10.1%) | 5 (10.0%) | 3 (12.5%) | 2 (16.7%) | 2 (20.0%) |
| 8 | 9 (7.4%) | 13 (9.1%) | 11 (7.6%) | 9 (8.0%) | 7 (7.9%) | 4 (8.0%) | 3 (12.5%) | 2 (16.7%) | 2 (20.0%) |
| 9 | 5 (4.1%) | 7 (4.9%) | 6 (4.1%) | 3 (2.7%) | 5 (5.6%) | 2 (4.0%) | 2 (8.3%) |  |  |
| 10 | 5 (4.1%) | 4 (2.8%) | 5 (3.4%) | 3 (2.7%) | 2 (2.2%) | 1 (2.0%) |  |  | 1 (10.0%) |
| Graft |  |  |  |  |  |  |  |  |  |
| HT | 103 (86.6%) | 124 (89.9%) | 127 (89.4%) | 100 (90.9%) | 80 (90.9%) | 45 (91.8%) | 23 (100.0%) | 12 (100.0%) | 10 (100.0%) |
| PT | 6 (5.0%) | 8 (5.8%) | 7 (4.9%) | 5 (4.5%) | 4 (4.5%) | 3 (6.1%) |  |  |  |
| Quadriceps | 5 (4.2%) | 4 (2.9%) | 5 (3.5%) | 4 (3.6%) | 4 (4.5%) | 1 (2.0%) |  |  |  |
| Allograft | 1 (0.8%) | 1 (0.7%) | 1 (0.7%) | 1 (0.9%) |  |  |  |  |  |
| Other | 4 (3.4%) | 1 (0.7%) | 2 (1.4%) |  |  |  |  |  |  |
| Side of graft harvest | |  |  |  |  |  |  |  |  |
| Contralateral | 7 (5.9%) | 11 (8.0%) | 10 (7.0%) | 10 (9.1%) | 7 (8.0%) | 4 (8.2%) | 2 (8.7%) | 1 (8.3%) | 2 (20.0%) |
| For categorical variables n (%) is presented. For continuous variables Mean (SD) / Median (Min; Max) / n= is presented. BMI= Body Mass Index; cm= centimetres; kg= kilogram; m= months; m2= 10 weeks follow-up; m^2^= metres squared; n= number; ^1^= Tegner Activity Scale. | | | | | | | | | |
